# Recurrence of Chromosome Rearrangements and Reuse of DNA Breakpoints in the Evolution of the Triticeae Genomes

**DOI:** 10.1534/g3.116.035089

**Published:** 2016-10-10

**Authors:** Wanlong Li, Ghana S. Challa, Huilan Zhu, Wenjie Wei

**Affiliations:** Department of Biology and Microbiology, South Dakota State University, Brookings, South Dakota 57007

**Keywords:** breakpoint reuse, chromosomal speciation, recurrent translocation, Triticeae, wheat

## Abstract

Chromosomal rearrangements (CRs) play important roles in karyotype diversity and speciation. While many CR breakpoints have been characterized at the sequence level in yeast, insects, and primates, little is known about the structure of evolutionary CR breakpoints in plant genomes, which are much more dynamic in genome size and sequence organization. Here, we report identification of breakpoints of a translocation between chromosome arms 4L and 5L of Triticeae, which is fixed in several species, including diploid wheat and rye, by comparative mapping and analysis of the draft genome and chromosome survey sequences of the Triticeae species. The wheat translocation joined the ends of breakpoints downstream of a *WD40* gene on 4AL and a gene of the *PMEI* family on 5AL. A basic helix-loop-helix transcription factor gene in 5AL junction was significantly restructured. Rye and wheat share the same position for the 4L breakpoint, but the 5L breakpoint positions are not identical, although very close in these two species, indicating the recurrence of 4L/5L translocations in the Triticeae. Although barley does not carry the translocation, collinearity across the breakpoints was violated by putative inversions and/or transpositions. Alignment with model grass genomes indicated that the translocation breakpoints coincided with ancient inversion junctions in the Triticeae ancestor. Our results show that the 4L/5L translocation breakpoints represent two CR hotspots reused during Triticeae evolution, and support breakpoint reuse as a widespread mechanism in all eukaryotes. The mechanisms of the recurrent translocation and its role in Triticeae evolution are also discussed.

Chromosome rearrangements (CRs), inversions and translocations in particular, underlie an important mechanism of karyotype evolution and postzygotic isolation, leading to adaptation to new environments and speciation (Rieseberg and Livingstone 2003; Ayala and Coluzzi 2005; Faria and Navarro 2010). It is generally believed that CR heterozygotes produce unbalanced gametes, which cause sterility and contribute to reproductive isolation, as has been well demonstrated in yeast (Hou *et al.* 2014). Alternatively, CRs may contribute to postzygotic isolation by suppressing recombination in rearranged fragments (Noor *et al.* 2001; Rieseberg 2001; Navarro and Barton 2003), as supported by enhanced diversity near CR breakpoints in sunflowers (Strasburg *et al.* 2009) and malaria mosquitos (Lee *et al.* 2013). In addition, CRs may provide selective advantage by creating new adaptation blocks (Joron *et al.* 2011), altering expression of genes at or in the vicinity of the CR breakpoints (Lister *et al.* 1993; Puig *et al.* 2004), or creating new chimeric genes (Hewitt *et al.* 2014). Therefore, precise localization of the CR breakpoints and determination of breakpoint structure are crucial for the elucidation of the molecular mechanisms underlying genome restructuring and chromosomal speciation.

The role of CRs in plant evolution has long been recognized (Stebbins 1958), and the Triticeae tribe is one of the best documented plant systems for chromosomal speciation studies. Triticeae contains several important cereal crops, including barley, rye, and wheat, which have coevolved for the last 11 MY (Figure 1). Rich genetic stocks and chromosome identification techniques make wheat (*Triticum* L.) and its close relatives, *i.e.*, goatgrass (*Aegilops* L.), a model for cytogenetic studies of polyploidy and chromosomal variation. Diploid species of *Triticum* and *Aegilops* diverged 1.4 MYA (Gornicki *et al.* 2014) and their genomes remain highly collinear except for a few CRs, such as a translocation between incipient 4L and 5L chromosome arms detected in diploid wheat *T. urartu* (genome AA) (King *et al.* 1994) and *T. monococcum* (genome A^m^A^m^) (Devos *et al.* 1995; Dubcovsky *et al.* 1996). Both *T. urartu* and *T. monococcum* are involved in the origin of polyploid wheat lineages. *T. urartu* (genome AA) pollinated *A. speltoides* (genome SS) in two separate hybridization events, giving rise to tetraploid wheat *T. turgidum* (genomes AABB) ∼0.7 MYA and *T. timopheevii* (genomes AAGG) ∼0.4 MYA (Gornicki *et al.* 2014). More recently, crosses between *T. turgidum* and *A. tauschii* (genome DD) produced common wheat or bread wheat (*T. aestivum*; genomes AABBDD); and hybridization between *T. timopheevii* and another diploid wheat, einkorn (*T. monococcum* subsp. *monococcum*), generated the second hexaploid wheat, *i.e. T. zhukovskyi* (genomes AAGGA^m^A^m^). As a result, the 4AL/5AL translocation descended from the A-genome donor species *T. urartu* into the polyploid wheat lineages, in which the rearranged 4A and 5A chromosomes suffered additional CRs. In the Emmer lineage, the 5AL segment, which was translocated to 4AL in diploid wheat, interchanged with 7BS, and pericentric (Naranjo *et al.* 1987; Naranjo 1990; Maestra and Naranjo 1999) and paracentric inversions occurred in 4A with their breakpoints surrounding the 4AL/5AL translocation junction on 4AL (Devos *et al.* 1995; Mickelson-Young *et al.* 1995). The *T. timopheevii* lineage carries two different cyclic translocation complexes involving 5AL/4AL/3AL and 6AS/1GS/4GS (Gill and Chen 1987; Jiang and Gill 1994; Maestra and Naranjo 1999).

**Figure 1:**
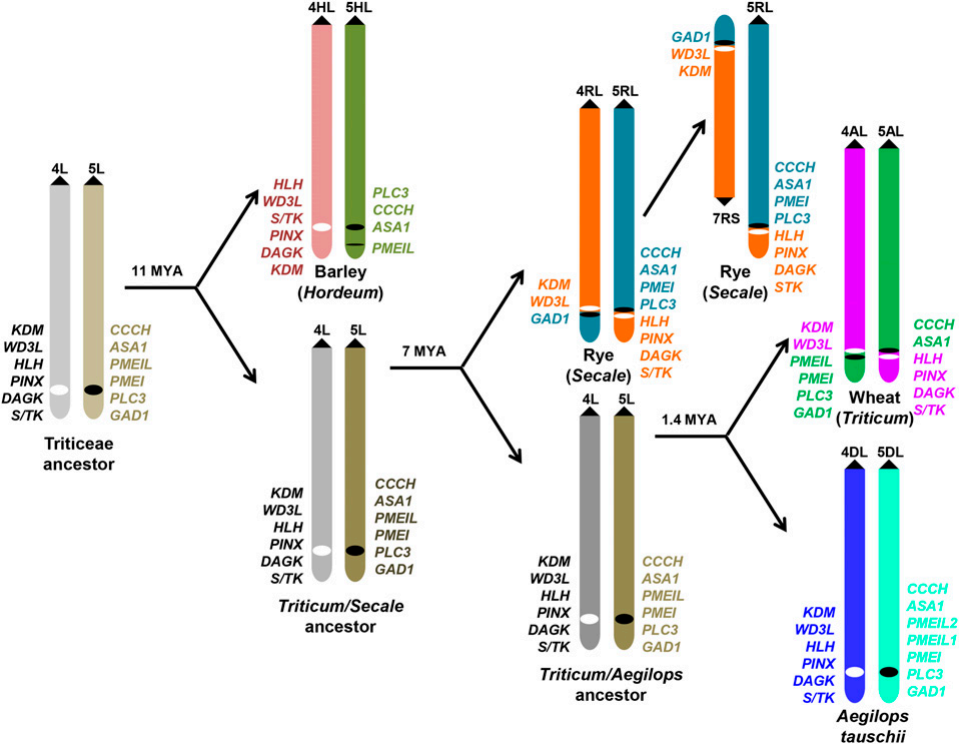
A diagram summarizing the evolutionary history of the distal regions of chromosome arms 4L and 5L of Triticeae diploids. Barley (H genome) diverged from the common ancestor of wheat and rye 11 MYA; rye (R genome) diverged from the common ancestor of wheat and goatgrass 7 MYA; and wheat (A genome) and *A. tauschii* (D genome) diverged 1.4 MYA. The dark filled triangles indicate the centromeres. The breakpoints and junctions of the 4L/5L translocation are indicated by the black and white dots. The genes flanking the translocation breakpoints and junctions are listed at the side in colors indicating their chromosome origin. The gene orders on 4HL and 5HL are tentative based on the barley draft genome sequences and the gene orders on 5RL and 7RS were deduced based on assumption of wheat-rye collinearity. The divergence time for the major Triticeae lineages was adopted from Huang *et al.* (2002) and Gornicki *et al.* (2014).

In addition to wheat, the 4L/5L translocations with similar breakpoint positions are also found in other species of the Triticeae tribe. Homoeologous chromosome pairing studies between wheat and rye (*Secale cereale* L.; genome RR) showed that the latter also carries this translocation by chromosomes 4R and 5R, and the rearranged 4RL arm was further translocated to 7RS (Naranjo *et al.* 1987; Naranjo and Fernández-Rueda 1991), which was further confirmed and characterized by comparative genome mapping (Liu *et al.* 1992; Rognli *et al.* 1992; Devos *et al.* 1993; King *et al.* 1994). Genotyping of wheat-alien addition lines using restriction fragment length polymorphism (RFLP) markers flanking the breakpoints detected this translocation on in *A. umbellulata* (genome UU) and *Thinopyrum bassarabicum* (genome E^b^ E^b^) (King *et al.* 1994), and the 4L/5L translocation in the former was further confirmed by genome mapping (Zhang *et al.* 1998). RFLP genotyping of wheat-*Haynaldia villosa* (genome VV) addition lines showed that three 4V addition lines and two of three 5V addition lines of different origins carried the translocation, suggesting the translocation is not fixed in this species (Qi *et al.* 1999). All of these findings suggest that the translocation occurred recurrently in Triticeae and that the translocation breakpoints are the two fragile sites on the chromosome arms 4L and 5L of diploid Triticeae species. These features make the 4L/5L translocations an attractive model for analysis of chromosomal speciation in plants.

Despite the importance of CRs in Triticeae evolution, molecular identification of the CR breakpoints in wheat and its relatives is confounded by the large genome size and high repeat content. To some extent, however, these problems have been alleviated by the recent progress in wheat genomics. The genome of *T. aestivum* cv. Chinese Spring (CS) (Brenchley *et al.* 2012), *A. tauschii* (Jia *et al.* 2013), and *T. urartu* (Ling *et al.* 2013) have been whole genome shotgun-sequenced. A parallel effort aims at sequencing the genomes of wheat and its relatives by map-based approaches. At the diploid level, bacterial artificial chromosome (BAC)-based physical maps have been constructed for the seven D-genome chromosomes of *A. tauschii* (Luo *et al.* 2013), based on which complete genome has been assembled (Dvorak *et al.* 2016). For polyploid wheat, chromosomes or chromosome arms from telosomic stocks of CS were flow-sorted based on their sizes and used for constructing BAC libraries (Safár *et al.* 2010) and shotgun sequencing (The International Wheat Genome Sequencing Consortium 2014). In addition, draft genome sequences are also made available for barley (*Hordeum vulgare* L.) (International Barley Genome Sequencing Consortium *et al.* 2012), and flow-sorted rye chromosomes have been survey-sequenced at low coverage (Martis *et al.* 2013). Combination of comparative mapping and comparative analyses of these genomic and chromosome sequence data enabled us to identify the breakpoints of the 4L/5L translocation with an unprecedentedly high resolution in Triticeae. Further comparative sequence analysis with the model grass genomes showed that the translocation breakpoints fall in the junction regions of ancient chromosome inversions in the Triticeae ancestor. Here, we report the results and implications of the recurrent CRs for plant genome evolution.

## Materials and Methods

### Plant materials

The plant materials used, and description and sources of seeds are listed in Supplemental Material, Table S1. Plants were planted in 2 × 2″ square pots containing Sunshine mix #3 (Sun Gro Horticulture, Agawama, MA) mixed with Perlite and Vermiculite (Hummurt International, St. Louis, MO), and supplied with a layer of controlled release Multicote fertilizer (Haifa Group, Haifa, Israel) and grown in a greenhouse, in which the temperature was 22° at day (16 hr) and 17° at night (8 hr).

### Genotyping and mapping

Genomic DNA of wheat species was isolated following the procedure described by Li *et al.* (2008), and DNA samples of a mapping population, consisting of 118 F_2_ individuals derived from a cross between AL8/78 and TA1604, were obtained from a previous project (Li and Gill 2006). PCR was set up in 10 µl, including ∼50 ng of DNA as a template, 1× GoTaq Reaction Buffer (Promega, Madison, WI), 250 µM dNTPs, 0.1 U Taq polymerase, and 1 μM primers (Table S2). For PCR analysis of BAC clones, 1 µl of overnight culture was used as a template. The PCR products were separated by electrophoresis in 1.2% agarose gels. The procedures described by Zhang *et al.* (2015) were followed for marker development, genotyping, and map construction.

### Transcription assay

Total RNA was isolated using TRIzol Reagent (Thermo Fisher Scientific, Inc., Waltham, MA), and cDNA was synthesized using QuantiTect Reverse Transcription Kit (Qiagen, Valencia, CA) following the manufacturers’ instructions. Approximately 10 ng of cDNA was used as template for reverse transcription (RT)-PCR. Primers used for PCR and RT-PCR are listed in Table S2.

### Sequence treatment and assembly

BAC clones of *A. tauschii* were obtained from Dr. Jan Dvorak, University of California (Davis, CA), and BAC DNA was isolated using BACMAX DNA Purification Kit (Epicentre, Madison, WI) following the manufacturer’s instruction. Selected BACs were sequenced by MiSeq platform (Illumina, San Diego, CA). The program Trimmomatic v0.30 (Bolger *et al.* 2014) was used for trimming the primer and adapter sequences, prinseq v0.20 (Schmieder and Edwards 2011b) for filtering low quality reads, and Deconseq v0.4.3 (Schmieder and Edwards 2011a) for removing reads that were derived from the BAC vector and *Escherichia coli* DNA. The clean reads were assembled using ABySS v1.3.6 (http://www.bcgsc.ca/platform/bioinfo/software/abyss) (Simpson *et al.* 2009) with *k*-mer of 75 or 89. Contigs from ABySS assemblies were assembled again to extend the contigs using CAP3 (Huang and Madan 1999) with an identity of 99% across 80-bp overlap. For filling the assembly gaps in the draft genome sequences, PCR products were dideoxy-sequenced from both ends, and the sequences were assembled using CAP3 program. All the genic sequences were deposited to GenBank under accessions numbers KX906948–KX906951.

### Sequence analysis

The D-genome marker sequences were retrieved from the D-genome marker database (http://probes.pw.usda.gov/cgi-bin/gb2/gbrowse/wheat_D_marker/), and physical distances between markers were estimated from the number of consensus fingerprinting bands to kilobases by multiplying a coefficient of 1.5 (Luo *et al.* 2013). *A. tauschii* BAC contig sequences were searched to validate location of the breakpoint genes in the D genome (Dvorak *et al.* 2016). The coding and protein sequences were deduced using the gene finding program, FGENESH (http://linux1.softberry.com/berry.phtml), with parameters set for monocot plants. After alignment with Triticeae repeats (http://wheat.pw.usda.gov/ITMI/Repeats/blastrepeats3.html), the non-transposable element (TE) coding sequences were used as queries for searching the assemblies of CS chromosome survey sequences and *T. monococcum* and *T. urartu* genomic sequences deposited in the central repository database (http://wheat-urgi.versailles.inra.fr/Seq-Repository). The non-TE protein sequences were used as queries for searching the *A. tauschii* and *T. urartu* draft genome sequences deposited in the nonredundant database of National Center for Biotechnology Information (NCBI) (http://blast.ncbi.nlm.nih.gov/Blast.cgi), the genome sequences of barley (082214v1) in the Ensembl plants database (http://plants.ensembl.org), the *Brachypodium distachyon* and sorghum genome sequences at the phytozome database (v10.1; http://phytozome.jgi.doe.gov/pz/portal.html), and the rice genome at the Rice Genome Annotation Project database (http://rice.plantbiology.msu.edu/) by BLASTP algorithm. The raw reads of rye chromosomes generated by Roche/454 platform (ERP001745) were retrieved from the Sequence Read Archive (SRA) of NCBI (http://www.ncbi.nlm.nih.gov/sra), and searched by a stand-alone BLASTN for the genes flanking the wheat breakpoints. Multiple sequence alignments were performed using the ClustalW program (http://www.genome.jp/tools/clustalw/).

For analyzing the distribution of poly(dA:dT) tracts, a custom python script was used to identify the tracts equal to or longer than 6 bp and retrieve their position and length information. The tract sequence density across a stretch of 100 bp (cumulative sum of the lengths of tracts present in the 100 bp stretch) was calculated and plotted against the position bin in the sequence

For localizing the breakpoints identified based on comparative sequence analysis onto the deletion bins of wheat chromosomes, the D-genome marker sequences flanking the breakpoints were used as queries to search the deletion bin-mapped Triticeae expressed sequence tags (ESTs) at GrainGenes website (http://wheat.pw.usda.gov/GG2/blast.shtml). To analyze the expression patterns of the breakpoint genes, NCBI EST database and RNA-Seq–based gene expression database of polyploid wheat, WheatExp (http://wheat.pw.usda.gov/WheatExp/), were searched using the BLASTN algorithm. Also, the raw reads of *T. urartu* (SRP005973) and *A. tauschii* (SRP005974) transcriptomes were downloaded from NCBI SRA, adaptor sequences were trimmed, and low quality reads were filtered out using the in-house pipeline mentioned above. The clean reads were mapped to the breakpoint genes using mapping tool available in CLCBio Genomics Workbench v6.5 (www.clcbio.com), and expression level is estimated in fragments per kilobase per million mapped fragments (FPKM).

### Data availability

The authors state that all data necessary for confirming the conclusions presented in the article are represented fully within the article.

## Results

Of the diploid progenitors of polyploid wheat, *T. monococcum* and *T. urartu* carry the 4L/5L translocation, but *A. speltoides* and *A. tauschii* do not have the translocation and instead have group 4 and group 5 chromosomes that are collinear with the common ancestor of the Triticeae tribe. Theoretically, alignment of the draft genome sequences of *A. tauschii* and *T. urartu* should reveal the translocation breakpoints. However, whole genome alignment is complicated by local gene loss and paralogous duplications, as well as assembly errors. To mitigate these difficulties, we adopted a map-based comparative genomics approach. We first anchored the 5L breakpoint position on 5DL by comparative mapping, then selected markers from that region on a 5DL physical map of *A. tauschii* to search the draft genome sequences of *A. tauschii* and *T. urartu*, and determined the chromosome arm locations of the genes in common wheat by BLASTN searches of the CS chromosome survey sequences, which led to identification of the 4AL junction of the 4AL/5AL translocation. The genes flanking the 4AL junction were, in turn, used as queries to search the 4DL physical map, CS chromosome survey sequences, and diploid wheat genome sequences, which provided more details of the 4AL breakpoint and 5AL junction of translocation. At the same time, PCR assays were employed to validate gene contexts. In this way, we anchored the translocation breakpoints onto the D genome, identified genes flanking the translocation breakpoints, and characterized the 4AL/5AL translocation junctions in the A and A^m^ genomes of wheat (Table S3). Using wheat breakpoint genes as queries, BLAST searches of the rye chromosome sequences and barley genomic sequences revealed the evolutionary history of these chromosome regions in the Triticeae tribe during the last 11 MY. Results summarizing the junction/breakpoint structure of 4L/5L translocations in relation to the Triticeae phylogeny are shown in Figure 1.

### Anchoring 5AL breakpoint of 4AL/5AL translocation to the D genome

During mapping of the *very short root 1* (*Vsr1*) locus, which is located on the 5DL segment collinear with the 5AL fragment translocated to 4AL (Li *et al.* 2013), we converted the RFLP markers flanking the 5AL breakpoint of the 4AL/5AL translocation into PCR-based markers and used them for genotyping an *A. tauschii* F_2_ population. The result showed that *XEsi4* and *XBG263925*, which are respectively proximal and distal to the 5AL breakpoint (Dubcovsky *et al.* 1996; Linkiewicz *et al.* 2004), cosegregated on 5DL (Figure S1). As the high-density linkage map and an anchored physical map were available for the D genome of *A. tauschii* (Luo *et al.* 2013), we aligned these maps based on sequence homology of the marker developed in the present research and those in the D-genome marker database and found that Esi4, which is homologous to CK210649, and BE446509 are contained in the extended sequences of markers AT5D5152 and AT5D5155 (Figure S1). We scanned the extended sequences of the markers surrounding loci *XAT5D5152* and *XAT5D5155* for their collinearity on homoeologous chromosomes in CS and anchored the diagnostic RFLP markers ABG391 and ABC310 for the 4AL/5AL translocation (Devos *et al.* 1995) to the flanking regions on 5DL (Figure S1). Most genes present in the 11 marker sequences from AT5D5142 to AT5D5152 are located on wheat chromosome arms 5AL, 5BL, and 5DL, and all genes in the 10 marker sequences from AT5D5153 to BE403637 are located on 4AL, 5BL, and 5DL in CS (Figure S1). All this indicates that markers AT5D5152 and AT5D5153 delimit the breakpoint of the 4AL/5AL translocation on the incipient 5AL chromosome arm. Further analysis of the extended sequences of 700 flanking markers, 614 proximal to *XAT5D5142* and 86 distal to *XBE403636*, on the 5D linkage map corroborated this conclusion.

**Figure 2:**
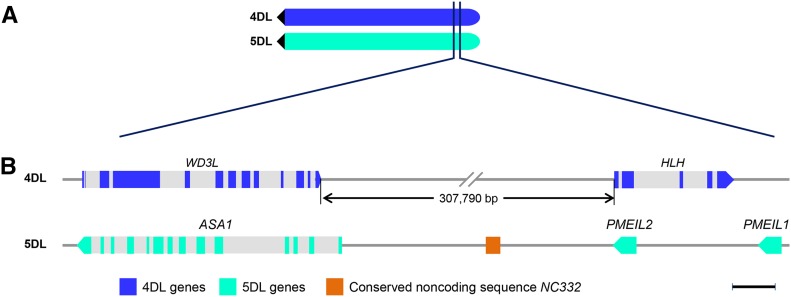
Sequence organization of chromosome arms 4DL and 5DL of *A. tauschii* corresponding to the 4L and 5L breakpoint regions. (A) A diagram of chromosome arms 4DL and 5DL showing the positions corresponding to the 4L and 5L breakpoints of the 4AL/5AL translocation in wheat. The dark filled triangle indicates the centromere. (B) Genes and sequence elements. The gene names are indicated at the top and the color codes are indicated at the bottom. In hexaploid wheat CS, *ASA1* is located on chromosome arms 5AL, 5BL, and 5DL but *PMEIL1* and *PMEIL2* are on 4AL, 5BL, and 5DL; *WD3L* is located on chromosome arms 4AL, 4BL, and 4DL but *HLH* is on 5AL, 4BL, and 4DL (Table S3). Scale bar, 1 kb.

BG263925 retrieved two genome scaffolds, one from 2DS and another from 5DL. The 5DL scaffold (GenBank accession no. KD524024) contains four genes encoding a zinc finger CCCH domain-containing protein 42 (CCCH; protein id EMT21622), a chloroplastic anthranilate synthase component α1 (ASA1; protein id EMT21623), a hypothetic protein (protein id EMT21624), and a putative plant invertase/pectin methylesterase inhibitor (PMEI; protein id EMT21625). EMT21624 showed 83% similarity to EMT21625, suggesting that it is a PMEI-like protein 1 (PMEIL1). Of these four genes, *CCCH* and *ASA1* detected homologs with high sequence identity on chromosome arms 5AL, 5BL, and 5DL and their homologs, with lower sequence identity on 2AS, 2BS, and 2DS in CS. However, *PMEIL1* and *PMEI*, which are homologous to BG263925, are located on chromosome arms 4AL, 5BL, and 5DL with low homology on 2BS and 2DS (Table S3). This result indicates that *ASA1* and *PMEIL1* delimited the 4AL/5AL translocation breakpoint on the incipient chromosome arm 5AL (Figure 2).

*ASA1* and *PMEIL1* are 8653 bp apart in KD524024. With the assembly gaps filled by sequencing the PCR products, a second *PMEI*-like gene, *PMEIL2*, was predicted from the 8653-bp interval. PMEIL2 protein showed 80 and 78% similarity to PMEIL1 and PMEI, respectively. In addition to their coding sequence, the flanking sequences of *PMEI*, *PMEIL1*, and *PMEIL2* also showed some similarity, suggesting that they were derived from direct gene duplication. A 332-bp conserved noncoding sequence 2102 bp proximal to the stop codon of *PMEIL2*, named *NC332*, detected homologs with >82% nucleotide identity on CS chromosome arms 4AL, 5AL, 5BL, and 5DL (Figure S2). Across the breakpoint, genes *ASA1*, *PMEIL2*, *PMEIL1*, and *PMEI* are all encoded by the opposite strand and transcribed in the centromere-bound direction on 5DL (Figure 2).

### 4AL junction of 4AL/5AL translocation

**Figure 3:**
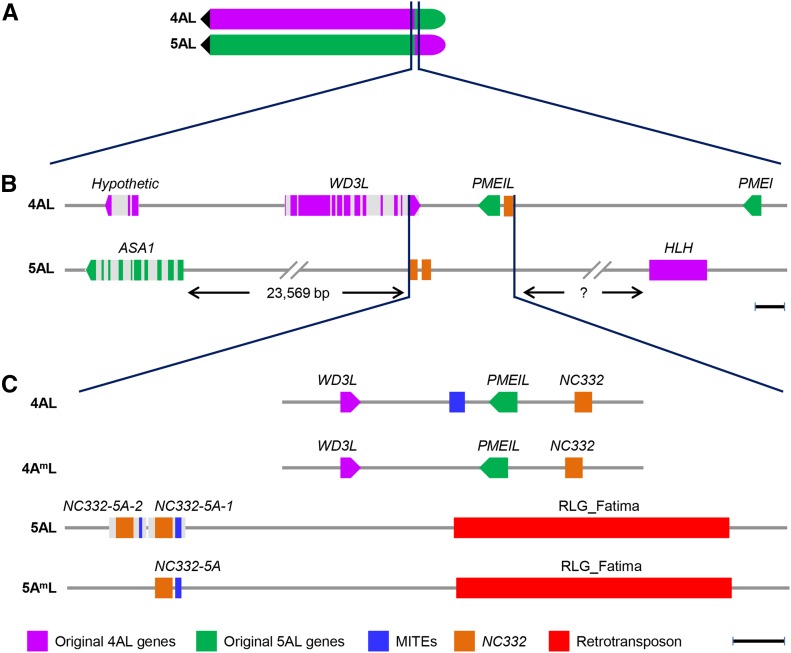
Structure of 4AL/5AL translocation junctions in wheat. (A) A diagram of chromosome arms 4AL and 5AL showing the positions of the 4AL/5AL translocation junctions. The dark filled triangles indicate the centromeres. (B) Genes across the translocation junctions. (C) Sequence organization at the translocation junction. The names of genes and elements are indicated above or under the drawings, and the color codes are indicated at the bottom. Scale bars, 1 kb.

Search of the CS chromosome sequences using the *PMEIL* gene as a query detected an A-genome homolog in CS sequence contig 4AL_7140977 with 90% identity. The 13,295-bp contig contains two genes coding for the PMEIL and a WD40 repeat-containing 3-like protein (WD3L). *PMEIL* and *DW3L* are encoded by two different stands and arranged in a tail–tail orientation. While *PMEIL*, as expected, is located on chromosome arms 4AL, 5BL, and 5DL, *WD3L* is located on 4AL, 4BL, and 4DL in CS, indicating *WD3L* and *PMEIL* define the 4AL/5AL translocation junction on 4AL (Figure 3B).

Analysis of the A-genome draft sequences of *T. urartu* corroborated this conclusion. Both *PMEIL* and *WD3L* colocalized in a genomic scaffold KD292409, which contains one pseudogene and five non-TE genes coding for proteins EMS44989–EMS44993. The genes for hypothetic protein 1 (EMS44989) and WD3L (EMS44990) are located on chromosome arms 4AL, 4BL, and 4DL, but genes for PMEI (EMS44991), phospholipase C 3 (PLC3; EMS44992) and hypothetic protein 2 (EMS44993) and a *glutamic acid decarboxylase 1* (*GAD1*) pseudogene are located on 4AL, 5BL, and 5DL (Figure 1, Figure 3, and Table S3). The sequence corresponding to *PMEIL* was not annotated in KD292409 due to a 261-bp assembly gap, and the *PMEIL* sequence in 4AL_7140977 was not complete either due to a 238-bp deletion. After filling the gap and deletion, we predicted the A-genome *PMEIL* gene for *T. urartu* and *T. aestivum*. Both *WD3L* and *PMEIL* were also found residing in a sequence contig of *T. monococcum*, contig_70829. The WD3L proteins encoded by the A and A^m^ genomes of the wheat species showed very high homology (Figure S3). High homology was also found among the A-genome *PMEIL* genes at nucleotide sequence level except a 13-bp deletion close to the 3′ end in CS, which caused a frame shift close to its C terminus (Figure S4). These results provide direct evidence that the 4AL/5AL translocation occurred in the common ancestor of *T. monococcum* and *T. urartu* before divergence of the A and A^m^ genomes and descended into polyploid wheat lineages through these diploid wheat species.

We validated the 4AL junction sequence, the interval delimited by the stop codons of *WD3L* and *PMEIL* by sequencing the PCR products and found that it is 2506, 2559, and 2554 bp long in *T. monococcum*, *T. urartu*, and *T. aestivum* cv. CS, respectively. The 4AL junction largely remains conserved except for a few small insertions/deletions (indels) (Figure S5). In *T. urartu*, a 706-bp sequence immediately downstream of (distal to) the stop codon of *WD3L* had similarity to sequences on chromosome arms 4AL, 4BL, and 4DL, and a 235-bp sequence immediately downstream of (proximal to) the stop codon of *PMEIL* had similarity to sequences on 4AL, 5BL, and 5DL. Part of these sequences match the ESTs of these two genes, indicating that they are the 3′ untranslated regions (UTR) of *WD3L* and *PMEIL*. As a result, the translocation junction on 4AL is narrowed down to 1618 bp in *T. urartu*, which is 4AL-specific and not collinear with the B or D genomes. A 265-bp Stowaway miniature inverted-repeat transposable element (MITE), homologous to DTT_Pluto_AY643844-1, is present in the A genome of *T. urartu* and *T. aestivum* but not in the A^m^ genome of *T. monococcum*, indicating that it inserted in the 4AL junction of *T. urartu* after divergence from *T. monococcum* and was transmitted to polyploid wheat. An 83-bp tandem duplication was detected in the proximal portion in *T. monococcum* but not in *T. urartu* and *T. aestivum* (Figure S5). As a result, the initial 4AL junction in the ancestor of *T. monococcum* and *T. urartu* was 1353 bp long. No *NC332* homologs were detected within the 2559-bp junction interval between *WD3L* and *PMEIL*; however, a homolog, *NC332-4A*, was found 1133 bp distal to the start codon of PMEIL and 7162 bp proximal to the stop codon of *PMEI* on 4AL (Figure 3).

**Figure 4:**
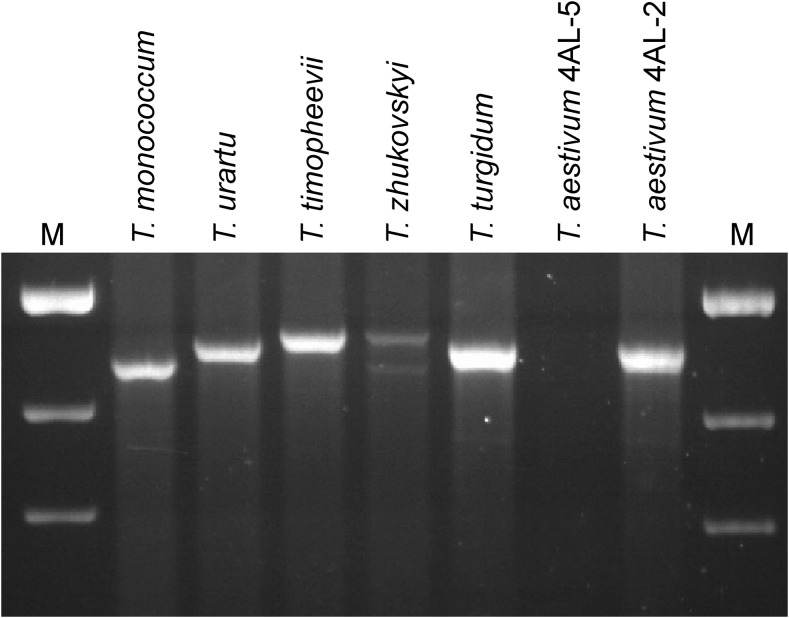
PCR assay of 4AL junction of the 4AL/5AL translocation in wheat species. Sources of DNA are indicated on the top of picture. *T. aestivum* 4AL-2 and 4AL-5 are deletion lines of chromosome arm 4AL in cultivar Chinese Spring, and their deletion breakpoint positions are indicated in Table S1. M: 1 kb ladder. Size of bands from the top: 3, 2, and 1.5 kb.

In addition to comparative sequence analyses among the wheat species, we also validated the 4AL junction of the 4AL/5AL translocation by PCR assay using a pair of primers binding to the 3′ UTR end of *WD3L* and the 3′ UTR of *PMEIL*. A single band of ∼2400 bp was amplified from all wheat species except *T. zhukovskyi*, which showed two bands corresponding to the A and A^m^ genome. The A-genome band is missing in CS deletion line 4AL-5 but present in 4AL-2, indicating its position in 4AL deletion bin 0.66–0.75, as expected (Figure 4). In addition, *WD3L* is also located in this deletion bin based on absence of amplicons in deletion lines 4AL-5, 4AL-7, 4AL-11, and 4AL-12, and presence of amplicons in 4AL-1 and 4AL-2 (Figure S6), while BG263925 was previously mapped to the same location by RFLP assays (Miftahudin *et al.* 2004).

### Anchoring 4AL breakpoint of 4AL/5AL translocation to the D genome

To identify the 4L breakpoint gene that was immediately distal to *WD3L* on the incipient 4AL and then translocated to modern 5AL, we searched the D-genome marker database using the *WD3L* genic sequence as a query and found a sole match in the extended marker sequence of AT4D4118. This marker anchors BAC contig ctg139, which contains two additional markers, AT4D4119 and BE638039, surrounding AT4D4118. Marker BE638039 is located on chromosome arms 4AL, 4BL, and 4DL in CS and is 308 kb proximal to AT4D4118 on 4DL (Figure S7). AT4D4119 is 876 kb distal to AT4D4118 and contains two genes coding for a diacylglycerol kinase catalytic domain-containing protein (DAGK) and a serine/threonine-protein kinase (S/TK). Both *DAGK* and *S/TK* detected homologs on chromosome arms 5AL, 4BL, and 4DL, indicating that the 4AL breakpoint is located in this 876-kb interval delimited by AT4D4118 and AT4D4119 (Figure S7).

*WD3L* retrieved the D-genome scaffold 57661 (KD556778), in which no additional genes were predicted. To narrow down the interval spanning the 4AL breakpoint, we sequenced AT4D4118-containing BACs HI297N22 and HI249E15 and two immediately distal BACs HD298C21 and HD560F06. From BAC HI297N22, a second gene, coding for a JmjC domain-containing lysine demethylase (KDM), was identified in addition to *WD3L* and the BAC sequence merged genomic scaffolds KD556778 and KD556692. *KDM* is located on CS chromosome arms 4AL, 4BL, and 4DL, and is 25,785 bp proximal to *WD3L* based on the merged genomic scaffold sequence. HD298C21 contains no gene, and HD560F06 contains a single gene that encodes a helix-loop-helix DNA-binding domain containing protein (HLH) and is 307,790 bp distal to *WD3L*. This result is further confirmed by *A. tauschii* BAC scaffold sequence 4627.1, which contains both *WD3L* and *HLH* and no other non-TE genes between them. *HLH* is located on the chromosome arms 5AL, 4BL, and 4DL in CS, indicating that *WD3L* and *HLH* define the boundaries of the 4AL/5AL translocation breakpoint on the incipient 4AL (Figure 2).

*S/TK* and *DAGK* are located in the D-genome scaffold 72003 (KD571076), in which *DAGK* was not annotated. The scaffold contains two additional genes, one coding for an F_box_assoc_1 domain-containing protein (FBA1) and another for a Pin2-interacting protein X1 (PINX1). Both *FBA1* and *PINX1* are located on chromosome arms 5AL, 4BL, and 4DL in CS (Table S3). PCR assays of these genes on BAC contig ctg139 indicated that the *PINX1-S/TK* interval is centromere-telomere oriented and distal to *HLH* (Figure S7).

### 5AL junction of 4AL/5AL translocation

**Figure 5:**
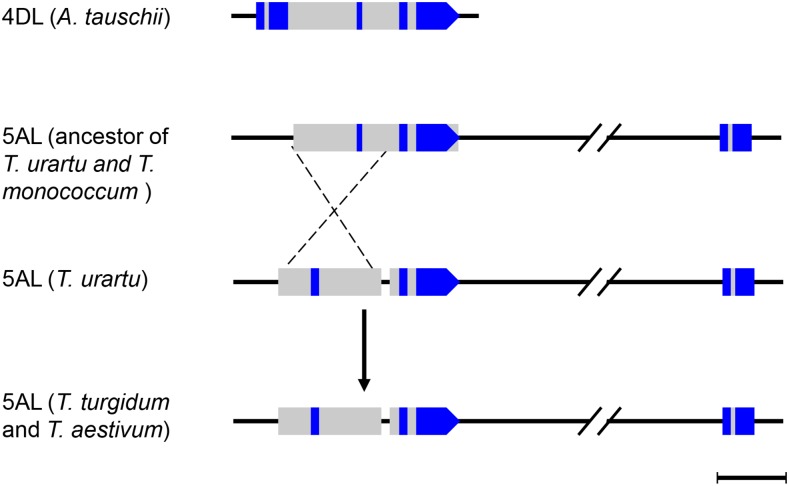
Comparative analysis of the *HLH* structure in the A, A^m^, and D genomes. Chromosome arms are indicated to the left of the figure and species are in parentheses. Exons are in blue, and introns are in light gray. Compared with the D genome, transposition of the first two exons to a distal region occurred in the ancestor of A and A^m^ genomes, and the third exon and second and third introns were subsequently inverted in the A genome. The orientation of the 5′ and 3′ fragments of the *HLH* gene on 5AL remains to be determined. The left end is toward centromere, and the right end is toward telomere. Scale bar, 1 kb.

On chromosome arm 5AL, the breakpoint genes from the incipient chromosome arms 4AL and 5AL are located in separate genomic scaffolds. Across the 5AL breakpoint in *T. urartu*, the genes encoding CCCH (EMS66758) and ASA1 (EMS66757) proteins are located in the genomic scaffold 32,617 (KD027639) and have the same orientation as in *A. tauschii* but *HLH* is split, with the first two exons located in the genomic scaffold 27,590 (KD212002) and the remaining part in the genomic scaffold 108,470 (KD244928). This intragenic split is confirmed in *T. monococcum*, *T. turgidum*, and *T. aestivum*. In addition to the 5′ part of HLH, KD212002 also contains a gene coding for MLO-like protein 1, the D-genome homolog of which is colocalized with marker AT4D4124 in KD579084, ∼4.3 Mb (4.3 cM) distal to the *HLH* gene in *A. tauschii*. This suggests that the 5′ part of *HLH* in the common ancestor of A and A^m^ genomes was transposed to a distal region on 5AL (Figure 5). *T. monococcum* is collinear with *A. tauschii* in the 3′ part of *HLH*, but an inversion involving the third exon and second and third introns occurred in *T. urartu* after divergence from *T. monococcum*, which was further transmitted to polyploid wheat (Figure 5). The transposition and intragenic inversion are validated by a PCR assay using the A genome–specific primers flanking its junction sequences, which produced single bands in CS but not in deletion line 5AL-23, hence localizing them to the 5AL deletion bin 0.87–1.00. *ASA1* is also located in the same deletion bin (Figure S6).

According to the annotation of KD027639, the 5′ portion of the *ASA1* gene in *T. urartu* is fused to the retrotransposon RLC_Inga. After an assembly gap was filled, however, we predicted a non-TE gene structure similar to its D-genome counterpart, which encodes a protein of 503 amino acids containing the Anth_synt_I_N domain at the N terminus and the Chorismate_bind domain at the C terminus (Figure S8). After sequence validation, a tandem duplication of *NC332* homolog (*NC332-5A*) was found 23,569 bp upstream of (distal to) the *ASA1* start codon, and both *NC332-5A* copies are associated with a Stowaway MITE that is homologous to DTT_Stolos_AY853252-1. Compared with the A genome of *T. urartu*, a single copy of NC332-5A is detected in this genomic region of the A^m^ genome of *T. monococcum* (Figure 3C), indicating that NC332-5A duplication occurred in *T. urartu* after divergence from *T. monococcum*. Because *NC332-5A* showed greater homology with the *NC332-5D* associated with *PMEIL2* on 5DL of *A. tauschii* as compared with the *NC332-4A*–associated with *PMEI* on 4AL (Figure S2), it is deduced that *NC332-5A* was associated with (proximal to) *PMEIL* on the incipient 5AL, and that *NC332-5A* remained in its position on the current 5AL when *PMEIL* was translocated to the current 4AL. Thus, the breakpoint on the incipient 5AL is located between *NC332-5A* and *PMEIL*.

No gene was predicted in the region 44 kb distal to *NC332-5A* in KD027639. Between the NC332-5A and the nearest TE, which is homologous to RLG_Fatima_AY494981-2, there is a 4690-bp 5AL-specific non-TE segment. These features are conserved in the A genome of *T. urartu* and *T. aestivum* and the A^m^ genome of *T. monococcum* (Figure S9). Further distal from the RLG_Fatima to the end of the scaffold sequence KD027639, the 41,670-bp sequence is full of nest-inserted TEs. Compared with the 4AL junction, the 5AL junction of the 4AL/5AL translocation is much larger and less well-defined (Figure 3C).

### Expression of the breakpoint genes in diploid ancestors of wheat

To gain insight into effect of the translocation on the transcription of the breakpoint genes, we mined the wheat transcriptome databases. We first searched the wheat ESTs deposited in NCBI and WheatExp, an expression database for polyploid wheat, and found that while *WD3L* and *ASA1* genes of the A, B, and D genomes were transcribed, the A- and D-genome copies of *PMEIL* were transcribed. As predicted, no transcript was found for the 5AL copy of *HLH*, but no match was found for the B- and D-genome copies either. We subsequently comparatively analyzed the RNA-seq dataset of *A. tauschii* and *T. urartu*, which are polymorphic for the 4L/5L translocation. Each transcriptome consists of ∼1.4 billion RNA-seq reads from a single biological replicate. Transcript quantification showed different patterns for the genes distal and proximal to the breakpoints between these two species (Figure S10). Two distal genes, *HLH* and *PMEIL*, were inactivated in *T. urartu*. *PMEIL* transcription reduced >1000-fold in *T. urartu* compared with *T. tauschii*; 17 reads of *A. tauschii* were mapped to exon 4 of *HLH*, but not a single read of *T. urartu* was mapped to its *HLH*. By contrast, transcription of proximal genes *ASA1* and *DW3L* only increased 2- and 1.5-fold, respectively, in *T. urartu* compared with *A. tauschii* (Figure S10). This result provided intriguing information for validation with additional RNA-seq data from multiple biological replicates for statistical robustness. Mapping of RNA-seq reads and RT-PCR assays using the A genome–specific primers indicated that only exons for the catalytic domains of the ASA1 protein, instead of the exons derived from the RLC_Inga, were transcribed (Figure S11), confirming the previous conclusion that the *ASA1* gene is not fused to RLC_Inga on 5AL.

### 4RL/5RL translocation breakpoints of rye

In addition to *Triticum*, the 4L/5L translocation is also present in several other Triticeae species including rye. If rye shares the breakpoints of the 4AL/5AL translocation, the 4L junction should be detected on current rye chromosome 7R and the 5L junction on 5R. We tested this hypothesis by searching the rye chromosome survey sequences using the wheat genes flanking the breakpoints as queries. We found that *KDM* and a majority of the *WD3L* gene body are located on 7R. Distal to *WD3L*, *HLH*, *PINX1*, and *FBA1* were translocated to 5R (Table S4). At the 5L side, *CCCH*, *ASA1*, *PMEI*, and *PLC3* detected orthologs on 5R, but *PMEIL* hit a homolog on chromosome 3R with the highest identity (89%) and *GAD1*, distal to *PLC3*, was found on 7R (Table S4). These results indicate that the 4RL/5RL translocation breakpoint on the incipient 4RL is between *WD3L* and *HLH*, and the breakpoint on the incipient 5RL is between *PLC3* and *GAD1* (Figure 1). Therefore, wheat and rye share the same position for the 4L breakpoint but not for the 5L breakpoint, although the positions of 5AL and 5RL breakpoints are very close.

### Collinearity interruption in barley

**Figure 6:**
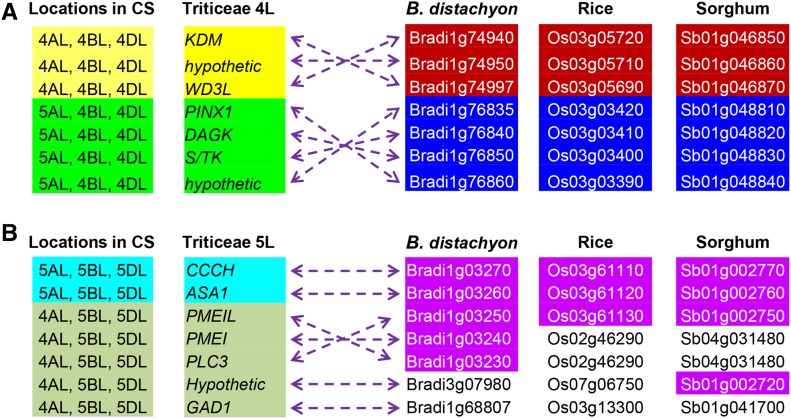
Microcollinearity of the breakpoint genes among the grass genomes. (A) Compared with the model grass genomes, the 4L breakpoint region of Triticeae went through two inversions. (B) Relative to *B. distachyon*, the 5L breakpoint region of Triticeae had one inversion. The collinear blocks in *B. distachyon*, rice, and sorghum are labeled with the same color. In Triticeae, the collinear blocks are divided by the 4AL/5AL breakpoints and color-labeled. The dashed arrow lines link the homologs in Triticeae and the model grasses.

Barley does not contain the 4L/5L translocation, but it would be intriguing to examine if the collinearity of the breakpoint genes is maintained between barley and *A. tauschii* after 11 MY of divergent evolution. Searches of the latest version of barley draft genome assembly found tentative CRs in these genomic regions (Figure 1 and Table S5). On 4HL, *KDM*, together with several genes from the collinear region, is located distal to *DAGK*. The order of the genes distal to the breakpoint, including *DAGK*, *FBA*, *HLH*, *PINX1*, and *S/TK*, is also changed on 4HL relative to 4DL (Table S5). Except for *FBA1*, *A. tauschii* genes in this genomic interval showed good collinearity with rice homologs (Figure 6A), indicating that tentative 4HL inversions occurred in the barley lineage of the Triticeae after divergence from the wheat/rye lineage (Table S5). Compared with *A. tauschii*, *HLH* orientation is inverted in barley. On 5HL, *CCCH* and *ASA1* remained collinear with the wheat orthologs, but *PMEI* was not detected in the barley genome while *PMEIL* is located ∼4 Mb distal to *ASA1*, and *PLC3* is located proximal to *CCCH* (Table S5). These results indicate that barley and *A. tauschii* are syntenic but may not be collinear in genomic regions corresponding to the 4L/5L translocation breakpoints, possibly due to small inversions or transpositions.

### Collinearity of breakpoint genes among grasses

For more detailed information on the evolutionary history of these genomic regions, we carefully analyzed the collinearity of the wheat breakpoint genes with the model grasses, rice, *B. distachyon*, and sorghum. The wheat genes flanking the 4AL breakpoint are collinear to two different genomic regions on the same chromosomes in all the model grass genomes, with the exception of *HLH* and *FBA1*, suggesting that these two genes were transposed to their current locations in the Triticeae lineage. The two genomic blocks were swapped in Triticeae, but the original orientation of gene orders remained within them. This finding indicates that this region experienced two paracentric inversions, and the 4L breakpoint coincides with the junction of these two inversions in the Triticeae ancestor after divergence from those model grasses (Figure 6A). The wheat genes across the 5L breakpoint showed good synteny with the top end of *B. distachyon* chromosome 1, where both *PMEI* and *PMEIL* are present, indicating that the *PMEI/PMEIL* duplication occurred in the common ancestor of *B. distachyon* and Triticeae 40 MYA. Despite the conserved gene synteny between *B. distachyon* and Triticeae across the 5L breakpoint, an inversion involving *PMEI*, *PMEIL*, and *PLC3* occurred in the Triticeae lineage (Figure 6B).

## Discussion

The CRs fixed at the species level appear to play a major role in speciation, and determining their breakpoint structure is the first and critical step to understanding their evolutionary mechanisms. Although evolutionary CR breakpoints have been cloned in model organisms, including yeast (Fischer *et al.* 2000), fruit flies (Prazeres da Costa *et al.* 2009), malaria mosquitos (Sharakhov *et al.* 2006), and primates (Goidts *et al.* 2005), little is known about the breakpoint structure of fixed CRs in plants at the sequence level, in part due to the much more complex and dynamic nature of their genomes. Using a map-based comparative genomics approach, we identified the breakpoints of the 4AL/5AL translocation fixed in wheat at the sequence level, which are corroborated by four independent genome assemblies of diploid wheat *T. monococcum* and *T. urartu* and polyploid wheat *T. aestivum*, and validated by PCR assays in all wheat species. Furthermore, we also determined breakpoint positions of the 4RL/5RL translocation in rye. These are the first breakpoints of an evolutionary translocation identified in plants, providing an opportunity to study mechanisms of this recurrent translocation and its role in the evolution of the Triticeae species.

### Recurrent translocation and breakpoint reuse

The 4L/5L reciprocal translocation was found in several Triticeae species with the breakpoint positions identical or very close, based on low-resolution genetic maps (King *et al.* 1994), which led to the conclusion that this translocation occurred in the common ancestor of wheat and rye (Martis *et al.* 2013). However, this hypothesis contradicts the Triticeae phylogeny, where *Aegilops* is more closely related to *Triticum* than to *Secale*, but many *Aegilops* species, including *A. speltoides* and *A. tauschii*, do not carry this translocation. An alternative scenario may be that the 4L/5L translocation occurred independently in the Triticeae lineages. Our analysis showed that the 4RL breakpoint in rye is collinear to the 4AL breakpoint in wheat, but the 5RL and 5AL breakpoints, although located nearby, are different, supporting an independent recurrence of the 4L/5L translocation in wheat and rye.

Chromosome arms 4DL and 5DL of *A. tauschii* are, in general, syntenic to 4HL and 5HL of barley (Jia *et al.* 2013). Alignment of the genes across the 4AL/5AL translocation breakpoints between the D and H genomes, however, revealed tentative inversions and possible transpositions. In fact, the breakpoint regions in the ancestral Triticeae genome are products of more ancient CRs, *i.e.*, inversions, on 4L and 5L, as revealed by alignment of the D-genome markers with the model grass genomes, including *B. distachyon*, rice, and sorghum, where all the model genomes are collinear to each other but not collinear to the Triticeae genomes (Figure 6). Taking together, all these findings indicate that the 4L/5L translocation breakpoints represent two CR hotspots in the Triticeae genomes and were reused during evolution of the Triticeae tribe.

As a relatively new hypothesis challenging the random breakpoint model (Ohno 1973; Nadeau and Taylor 1984), breakpoint reuse was proposed for human and mouse genomes (Pevzner and Tesler 2003), and is now gaining support from yeast (Hewitt *et al.* 2014) and *Drosophila* (Puerma *et al.* 2014) researchers as well. Our results from plant genomes support breakpoint reuse as a widespread mechanism in all eukaryotes.

### Molecular mechanisms for recurrent CR occurrence

What makes the recurrent CR breakpoints so susceptible to rearrangement? The uniqueness of local genomic/epigenomic landscapes would be a possible answer. In primates, the breakpoints of a recurrent inversion in chimpanzee and gorilla chromosomes, which are homologous to human chromosome 16, are near a conserved 23-kb inverted repeat composed of satellites, LINE, and Alu elements. It is believed that this repeat mediated the inversion by bringing the chromosomal arms into close proximity, hence facilitating intrachromosomal recombination (Goidts *et al.* 2005). In the present study, breakpoints of 4AL/5AL translocation are close to the 3′ end of the genes by end-joining, as demonstrated by the 4AL junction, suggesting that the local genomic or epigenomic landscape played a role in double-strand break (DSB) formation (Figure 3, Figure S5, and Figure S8). In *Arabidopsis*, poly(dA:dT) tracts are the dominant motifs of scaffold/matrix-associated regions (S/MARs) (Pascuzzi *et al.* 2014), and the S/MARs are harbored in chromosome fragile sites (dela Paz *et al.* 2012). We also found poly(dA:dT) tracts at frequencies of 18.4/kb in the 4AL junction and 19.6/kb in the 5AL junction, which are slightly higher than the average frequencies for chromosome 4A (11.6/kb) and chromosome 5A (14/kb). It would be desirable to test if the breakpoint regions are frequently colocalized within the same nuclear territory using the Hi-C technology (van Berkum *et al.* 2010).

Compared with the 4AL junction region, the 5AL junction region is much larger and TE-rich. *HLH* lost its first two exons in the common ancestor of *T. monococcum* and *T. urartu*, followed by an inversion of its third exon and second intron in the latter (Figure 5). These results imply that the translocation may have triggered secondary restructuring events or facilitated their accumulation at the initial 5AL junction, which obscured the structure of the original translocation junction.

### Possible role of 4L/5L translocation in Triticeae speciation

It is generally believed that fixation of a CR with strong underdominance can only happen in a small population by genetic drift in a self-pollinated species (Rieseberg 2001). As an exception, the 4RL/5RL translocation is fixed in rye (Khush 1963), which is a cross-pollinating species. As a recurrent CR, the 4L/5L translocation was not mediated by nonallelic TE recombination, a prevailing mechanism underlying CRs. These features suggest that 4L/5L translocation may cause overdominance leading to its selection (fixation). What contributed to the CR overdominance? Alteration of gene expression at or in the vicinity of the breakpoints caused by the CRs is a possible explanation. The genes flanking the 4AL/5AL translocation breakpoints are involved in many important biological processes and interactions with the environment. *WD3L* ortholog in *Arabidopsis* is required for exportation of mRNA out of and importation of proteins into nuclei; *HLH* belongs to the bHLH superfamily, involving many developmental processes and stress response (Carretero-Paulet *et al.* 2010); *ASA1* encodes the first enzyme for biosynthesis of phytohormone auxin (Mano and Nemoto 2012); and *PMEIL* is involved in multiple developmental programs, such as germination, by regulating cell wall homeostasis (Saez-Aguayo *et al.* 2013) and resistance to fungal and virus pathogens (Lionetti *et al.* 2007, 2014). Analysis of single transcriptomes of the A- and D- genome progenitors showed that distal breakpoint genes *HLH* and *PMEIL* are inactivated but proximal breakpoint genes *ASA1* and *WD3L* are slightly upregulated in *T. urartu* (Figure S10). Further investigation is needed to validate the differences, using multiple biological replicates from collinear and rearranged genome, and to determine whether the translocation also caused any alterations in tissue and cell-type specificity of the expression.

An alternative explanation may also be possible, where transcriptional change of the breakpoint genes was not due to the translocation, but the 4L/5L translocations in wheat and rye created new linkage blocks, or supergenes, of adaptation. In the mimetic butterfly, a set of coadapted genes for mimicry was assembled by an inversion (Joron *et al.* 2011). This “supergene” model also explains why some species do not carry this recurrent translocation because the presence of the 4L/5L translocation not only depends on the frequencies of DSBs at both breakpoints, which is related to the local genomic and epigenomic landscape, but also depends on alleles of genes flanking the breakpoints. With more Triticeae genomes sequenced, this hypothesis can be tested in wheat and its relatives by characterizing the genes surrounding the 4L/5L translocation junctions for their homoallelic variation, pathways, and regulation in comparative and functional genomics approaches. *H. villosa* is another cross-pollinating Triticeae species in which the 4VL/5VL translocation has not been fixed (Qi *et al.* 1999). Association mapping in the breakpoint regions may lead to identification of supergene components and the traits that they control.

CRs also can contribute to postzygotic isolation via suppressing recombination in the rearranged fragments, and suppression of recombination in turn fosters the accumulation of gene mutations (Noor *et al.* 2001; Rieseberg 2001; Navarro and Barton 2003). In our study, we found that CRs also facilitate gene insertions. At and in the vicinity of the 4L breakpoint, *HLH* and *FBA1* are collinear among the Triticeae species but not collinear with the model grass genomes, suggesting that they were probably transposed to their current position after the ancient 4L inversions occurred in the Triticeae ancestor. Transposition often leads to duplication, and duplicated genes show an elevated mutation rate due to selection relaxation for neofunctionalization or subfunctionalization (Barker *et al.* 2012). Some of the mutations including new genes may eventually help establish genetic incompatibility for postzygotic isolation between species polymorphic for the CRs, such as the A-genome donor species *T. urartu* and the B-genome donor species *A. speltoides*, and this incompatibility poses a bottleneck for polyploid wheat speciation and may be overcome by further CRs surrounding the 4AL junction in the polyploid wheat lineages. We are identifying the genes flanking the polyploid wheat-specific CR breakpoints. Molecular characterization of their structure may provide clues to how 4L/5L translocation contributed to Triticeae evolution.

### Conclusions

In this study, we identified the first breakpoints of an evolutionary translocation in plant genomes at the molecular level and demonstrated that the 4L/5L translocation breakpoints are two CR hotspots in Triticeae genomes. The translocation in wheat joined the ends of breakpoints downstream of a *WD40* gene on 4AL and a gene of the *PMEI* family on 5AL, and significantly restructured a basic helix-loop-helix transcription factor gene distal to the 4AL breakpoint. We also showed that the 4L/5L translocation recurrently occurred in evolution of Triticeae species with similar but not identical breakpoint structure in wheat and rye, and that the 4L/5L breakpoints coincide with the junctions of ancient inversions. Our results support breakpoint reuse as a widespread mechanism in all eukaryotes, including plants, and invite more studies on molecular mechanisms of genome instability and roles of CRs in plant evolution.

## Supplementary Material

Supplemental Material
