# Supplementary material for: Recurrence of Chromosome Rearrangements and Reuse of DNA Breakpoints in the Evolution of the Triticeae Genomes
Source: G3 (Bethesda). 2016 Oct 10;6(12):3837–47. doi: 10.1534/g3.116.035089 (PMC5144955; doi:10.1534/g3.116.035089)
Supplement: Supplemental Material [file supp_6_12_3837__index.html]

Recurrence of Chromosome Rearrangements and Reuse of DNA Breakpoints in the Evolution of the Triticeae Genomes — Supplemental Material 

# Recurrence of Chromosome Rearrangements and Reuse of DNA Breakpoints in the Evolution of the Triticeae Genomes

## Supplemental Material for Li *et al.*, 2016

**Files in this Data Supplement:**

- Figure S1 - Comparative mapping of 5AL breakpoint of the 4AL/5AL translocation in *A. tauschii*. (.pdf, 102 KB)
- Figure S10 - Transcription analysis of the breakpoint genes in *A. tauschii* and *T. Urartu*. (.pdf, 22 KB)
- Figure S11 - Transcription analysis of the A-genome *ASA1*. (.pdf, 59 KB)
- Table S1 - Chromosome arm locations of genes flanking the 4AL/5AL translocation breakpoints in wheat and its ancestors. (.pdf, 27 KB)
- Table S2 - Chromosome location of the breakpoint genes in the rye genome. (.pdf, 12 KB)
- Table S3 - Positions of breakpoint genes on barley chromosome arms 4HL and 5HL. (.pdf, 91 KB)
- Table S4 - A list of plant materials used. (.pdf, 92 KB)
- Table S5 - Primers developed by the present research for PCR and RT-PCR assays. (.pdf, 165 KB)
- Figure S2 - Sequence alignment of *NC332* homoeologs located in 4AL, 5AL, 5BL and 5DL chromosome arms of *T. aestivum* cv. CS. (.pdf, 73 KB)
- Figure S3 - Alignment of WD3L protein sequences of the A genomes of the genus *Triticum*. (.pdf, 50 KB)
- Figure S4 - Alignment of *PMEIL* cds (top) and predicted protein sequences (bottom) from the A genomes of the genus *Triticum*. (.pdf, 50 KB)
- Figure S5 - Sequence alignment of the 4AL junctions of the 4AL/5AL translocation from *T. monococcum*, *T. Urartu* and *T. aestivum*. (.pdf, 167 KB)
- Figure S6 - Deletion mapping of breakpoint genes by PCR using the A genome-specific primers. (.pdf, 75 KB)
- Figure S7 - Determination of the gene order in BAC contigs ctg139 by PCR assays. (.pdf, 107 KB)
- Figure S8 - Sequence alignment of proteins ASA-A1, ASA-B1 and ASA-D1 from the *ASA1* homeologous genes on 5AL of *T. Urartu*, 5BL of *T. aestivum* cv. Chinese Spring and 5DL of *A. tauschii*, respectively. (.pdf, 52 KB)
- Figure S9 - Sequence alignment of the 5AL junctions of the 4AL/5AL translocation from *T. monococcum*, *T. Urartu* and *T. aestivum*. (.pdf, 148 KB)
