# Supplementary material for: Recurrence of Chromosome Rearrangements and Reuse of DNA Breakpoints in the Evolution of the Triticeae Genomes
Source: G3 (Bethesda). 2016 Oct 10;6(12):3837–47. doi: 10.1534/g3.116.035089 (PMC5144955; doi:10.1534/g3.116.035089)
Supplement: Supplemental Material [file supp_g3.116.035089_FigureS1.pdf]

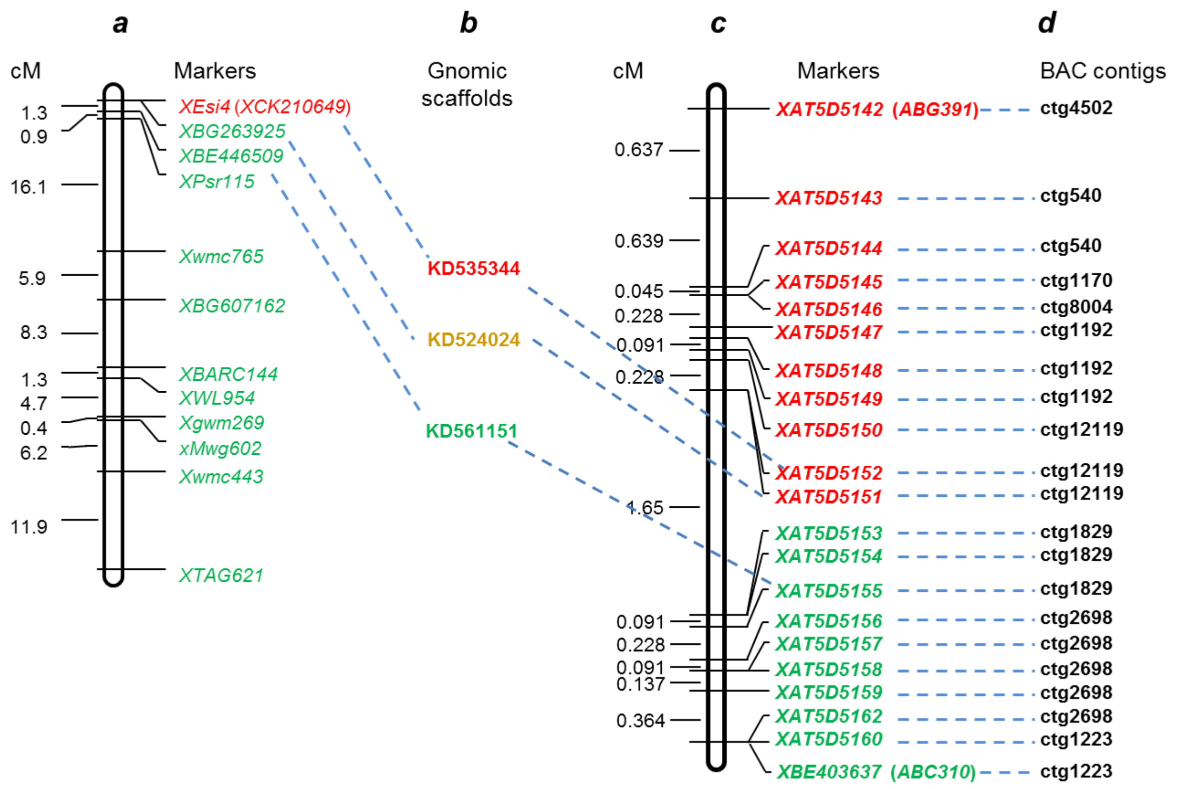

Figure S1. Comparative mapping of 5AL breakpoint of the 4AL/5AL translocation in *A. tauschii*. (***a***) Part of 5DL linkage map developed by the present research. (***b***) Genbank accession numbers of genomic scaffolds containing the marker sequences listed in (***a***). (***c***) A portion of 5DL high-density linkage map drawn based on mapping data from Lou et al (2013). The extended marker sequences were localized to CS chromosome arms by BLAST searches. The top of the maps is toward centromere and the bottom toward the telomere. At the right of the maps are the marker loci, and the left of the maps are genetic distances in centi-Morgan (cM). The loci in red contain genes with homologues on chromosome arms 5AL, 5BL and 5DL of polyploid wheat, marker loci in green contain genes with homologues on 4AL, 5BL and 5DL, and RFLP markers ABG391 and ABC310 are included in parentheses. The genomic scaffolds (***b***) are also color-coded, and the brown color indicates proximal part of KD524024 is located on chromosome arms 5AL, 5BL and 5DL, and distal part on 4AL, 5BL and 5DL. Dashed lines link the cognate markers and genomic scaffolds.
