# Supplementary material for: Recurrence of Chromosome Rearrangements and Reuse of DNA Breakpoints in the Evolution of the Triticeae Genomes
Source: G3 (Bethesda). 2016 Oct 10;6(12):3837–47. doi: 10.1534/g3.116.035089 (PMC5144955; doi:10.1534/g3.116.035089)
Supplement: Supplemental Material [file supp_g3.116.035089_FigureS10.pdf]

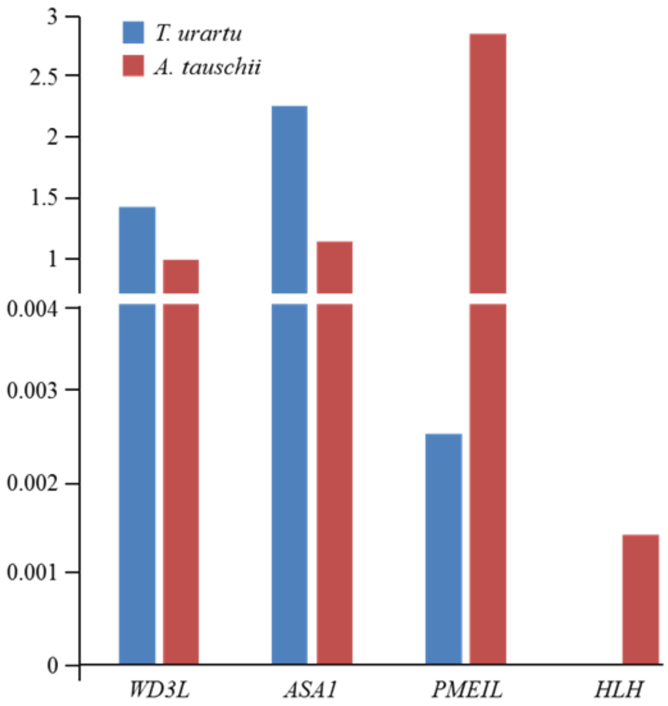

Figure S10. Transcription analysis of the breakpoint genes in *A. tauschii* and *T. urartu*. The clean and paired RNA-seq reads were mapped to the exons of the genes, counted and normalized. The gene names are indicated under the X axis and the expression levels are indicated on the Y axis in FPKM.
