# Supplementary material for: Recurrence of Chromosome Rearrangements and Reuse of DNA Breakpoints in the Evolution of the Triticeae Genomes
Source: G3 (Bethesda). 2016 Oct 10;6(12):3837–47. doi: 10.1534/g3.116.035089 (PMC5144955; doi:10.1534/g3.116.035089)
Supplement: Supplemental Material [file supp_g3.116.035089_FigureS11.pdf]

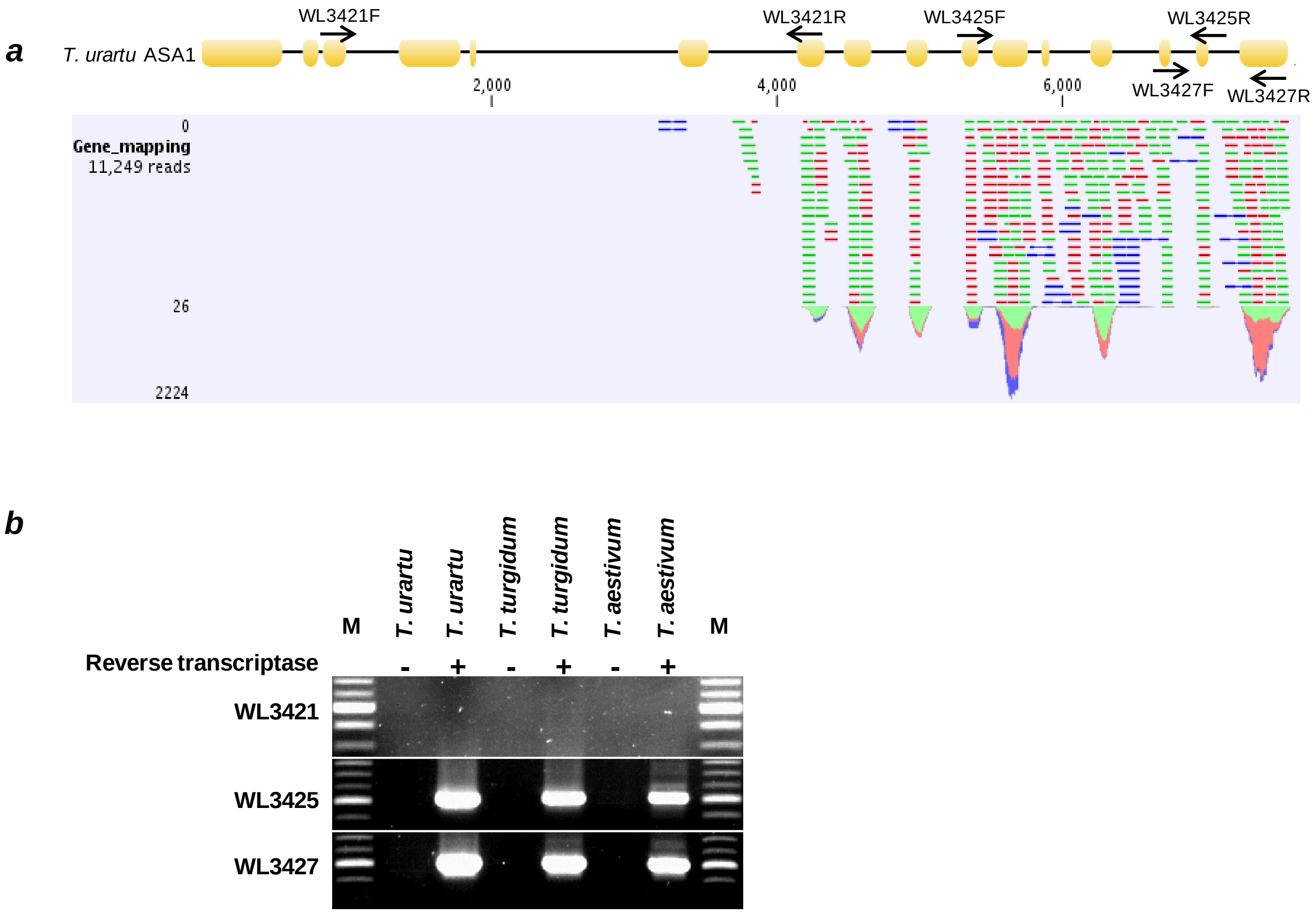

Figure S11. Transcription analysis of the A-genome *ASA1*. (***a***) Mapping of RNA-seq reads from the transcriptome of *T. urartu* to the ASA1 gene model (protein id EMS66757) at the top. The yellow blocks are the exons and the black line segments represent introns. The mapped reads are represented as green for the forward, red for the reverse and blue for the paired reads. (***b***) RT-PCR assays of the A genome copy of the *ASA1* gene in diploid, tetraploid and hexaploid wheat species. The species are indicated on the tope, and the markers are indicated in the left of the figure. Positions of the primer binding sites are indicated on the gene model, and the primer sequences are listed in Table S4. M: 100-bp ladders. The bright bands indicate 500 bp.
