# Supplementary material for: Recurrence of Chromosome Rearrangements and Reuse of DNA Breakpoints in the Evolution of the Triticeae Genomes
Source: G3 (Bethesda). 2016 Oct 10;6(12):3837–47. doi: 10.1534/g3.116.035089 (PMC5144955; doi:10.1534/g3.116.035089)
Supplement: Supplemental Material [file supp_g3.116.035089_TableS1.pdf]

Table S4. A list of plant materials used.

Species	Accessions	Description	Genome	Fraction length*	Sources
T. urartu	PI 428198	G1812	AA		Harold E. Bockelman
T. monococcum	TA192		A^mA^m		Bikram S. Gill
T. turgidum	TA3028	Langdon	AABB		Bikram S. Gill
T. aestivum	TA3008	Chinese Spring	AABBDD		Bikram S. Gill
T. aestivum	TA3278	N4A-T4D**	AABBDD		Bikram S. Gill
T. aestivum	TA3063	N5A-T5D**	AABBDD		Bikram S. Gill
T. aestivum	TA4528 L2	4AS-2 deletion line	AABBDD	0.71	Bikram S. Gill
T. aestivum	TA4529 L3	4AS-3 deletion line	AABBDD	0.76	Bikram S. Gill
T. aestivum	TA4529 L1	4AL-1 deletion line	AABBDD	0.85	Bikram S. Gill
T. aestivum	TA4530 L2	4AL-2 deletion line	AABBDD	0.75	Bikram S. Gill
T. aestivum	TA4531 L5	4AL-5 deletion line	AABBDD	0.66	Bikram S. Gill
T. aestivum	TA4532 L7	4AL-7 deletion line	AABBDD	0.66	Bikram S. Gill
T. aestivum	TA4533 L11	4AL-11 deletion line	AABBDD	0.66	Bikram S. Gill
T. aestivum	TA4534 L12	4AL-12 deletion line	AABBDD	0.43	Bikram S. Gill
T. aestivum	TA4535 L23	5AL-23 deletion line	AABBDD	0.87	Justin D. Faris
T. timopheevii	TIM01		AAGG		Moshe Feldman
T. zhukovskyi	PI 355706		$AAGGA^mA^m$		Harold E. Bockelman

* The position of deletion breakpoint was expressed as a fraction length value of the arm retained in the deletion chromosome.
**N4A-T4D and N5A-T5D are abbreviation of nullisomic 4A-tetrasomic 4D and nullisomic 5A-tetrasomic 5D.
