# Supplementary material for: Recurrence of Chromosome Rearrangements and Reuse of DNA Breakpoints in the Evolution of the Triticeae Genomes
Source: G3 (Bethesda). 2016 Oct 10;6(12):3837–47. doi: 10.1534/g3.116.035089 (PMC5144955; doi:10.1534/g3.116.035089)
Supplement: Supplemental Material [file supp_g3.116.035089_TableS2.pdf]

**Table S5.** Primers developed by the present research for PCR and RT-PCR assays

| Primers | Forward (5’→3’) | Reverse (5’→3’) | Description |
|---|---|---|---|
| WL909 | GGCGTTTTATACGGAGATGC | TTGCTACCGAGACACATTGG | PCR marker for BE446509, digested by HpyCH4IV |
| WL913 | CAAGCTCGCCGCCGTCGAG | CGACAAAAGACGTATAGACAAC | PCR marker for Esi4/CK210649, digested by NdeII |
| WL928 | TCTCTGAATCCCATCCATC | TCGAAGTACTGCGTGTAGCC | PCR marker for BG607162, digested by AluI |
| WL3391 | AAGAAACCCAAGGCGTCTGT | CATTTGATGTCCAGCGTGTG | 4AL junction for Figure 4 |
| WL3421 | AGCTCAATGCCAACACAATG | TGAGGTGGTCGGAGAAGATG | RT PCR targeting Inga-*ASA1* junction |
| WL3425 | GGCTCAGCGCTACAAAAATC | CATCACACAGCTCCCCAGTA | RT PCR targeting catalytic region of A-genome *ASA1* |
| WL3427 | AGGTGTCCAAACCAGGAACA | TGCCAGAGAAATCCAGGAAC | RT PCR targeting catalytic region of A-genome *ASA1* |
| WL3429 | GATGCCCATATTGGCTCCTA | CATGACTATGCCCCTCCATC | A genome-specific *ASA1*, PCR |
| WL3463 | GACCTTGGTTGTGGCTGACT | GCATGCGTAGTGTGAATGCT | 4AL junction, PCR |
| WL3467 | TCGACCTCCACCTTGAACTC | AAGCTTCCGCCTCTTCTTTC | *S/TK, A. tauschii*, PCR |
| WL3469 | AGGGGATGCTCTCTGGATCT | GCCTCAACCTTCTGTTCGAC | *FBA1, A. tauschii*, PCR |
| WL3471 | ATCCCTGATGTTAGGCGTTG | GGGGCACAAGTTTGGATATG | *PINX1, A. tauschii*, PCR |
| WL3475 | TCGTCCCCTTTTCCTGCTCC | CCCATTTGTTCCAATCTTCG | A genome-specific *WD3L*, PCR |
| WL3537 | CAGCTTCAGGGTTTTCTTGG | GGTTTGGAGACCGATTCAGA | *WD3L, A. tauschii*, PCR |
| WL3539 | GAGCAATTCCCTTCACGAAC | AAACATAGTGCCCGACATGC | A genome-specific *HLH*, PCR |
| WL3541 | CGTCTCGCCACTCAGATACA | GTAAGGAGGGTACGGGAACA | *HLH, A. tauschii*, PCR |
| WL3086 | TCTTTACAATTTTTTATGTTTTCAT | CACATATTTGTAAAATGGTTGTTGC | PCR marker for BG263925, digested by NlaIII |
