# Supplementary material for: Recurrence of Chromosome Rearrangements and Reuse of DNA Breakpoints in the Evolution of the Triticeae Genomes
Source: G3 (Bethesda). 2016 Oct 10;6(12):3837–47. doi: 10.1534/g3.116.035089 (PMC5144955; doi:10.1534/g3.116.035089)
Supplement: Supplemental Material [file supp_g3.116.035089_TableS3.pdf]

Table S1. chromosome arm locations of genes flanking the 4AL/5AL translocation breakpoints in wheat and its ancestors

| Species | Genome | Scaffolds | Genes | Protein id | Chromosome arms location in CS* |
|---|---|---|---|---|---|
| *A. tauschii* | D | KD524024 | *CCCH* | EMT21622 | 5AL, 5BL, 5DL, 2AS, 2BS, 2DS |
| | | KD524024 | *ASA1* | EMT21623 | 5AL, 5BL, 5DL, 2AS, 2DS |
| | | KD524024 | *PMEIL* | EMT21624 | 4AL, 5BL, 5DL, 2BS, 2DS |
| | | KD524024 | *PMEI* | EMT21625 | 4AL, 5BL, 5DL |
| | | KD505953 | *PLC3* | EMT30190 | 4AL, 5BL, 5DL |
| | | KD505953 | *hypothetic* | EMT30189 | 4AL, 5BL, 5DL |
| *A. tauschii* | D | KD556692 | KDM | EMT10917 | 4AL, 4BL, 4DL |
| | | KD556778 | *WD3L* | EMT10888 | 4AL, 4BL, 4DL |
| | | | *HLH* | | 5AL, 4BL, 4DL |
| | | KD571076 | *PINX1* | EMT07875 | 5AL, 4BL, 4DL |
| | | KD571076 | *FBA1* | EMT07874 | 5AL, 4BL, 4DL |
| | | KD571076 | *DAGK* | | 5AL, 4BL, 4DL |
| | | KD571076 | *S/TK* | EMT07873 | 5AL, 4BL, 4DL |
| | | KD571076 | *hypothetic*[¶] | | 5AL, 4BL, 4DL |
| *T. urartu* | A | KD212666 | KDM | EMS52005 | 4AL, 4BL, 4DL |
| | | KD292409 | *hypothetic1* | EMS44989 | 4AL, 4BL, 4DL |
| | | KD292409 | *WD3L* | EMS44990 | 4AL, 4BL, 4DL |
| | | KD292409 | *PMEIL* | | 4AL, 5BL, 5DL, 2BS, 2DS |
| | | KD292409 | *PMEI* | EMS44991 | 4AL, 5BL, 5DL, 2BS, 2DS |
| | | KD292409 | *PLC3* | EMS44992 | 4AL, 5BL, 5DL |
| | | KD292409 | *hypothetic2* | EMS44993 | 4AL, 5BL, 5DL |
| | | KD292409 | *GAD1*[§] | | 4AL, 5BL, 5DL, 4BL, 4BS, 4DL |
| *T. urartu* | A | KD027639 | *CCCH* | EMS66758 | 5AL, 5BL, 5DL, 2DS, 2BS, 2AS, |
| | | KD027639 | *ASA1* | EMS66757 | 5AL, 5BL, 5DL |
| | | KD244928 | *HLH* | | 5AL, 4BL, 4DL |
| | | KD162539 | *PINX1* | EMS56124 | 5AL, 4BL, 4DL |
| | | KD065313 | *DAGK* | EMS63785 | 5AL, 4BL, 4DL |

| | | | | | |
|---|---|---|---|---|---|
| | | KD065313 | *S/TK* | | 5AL, 4BL, 4DL |
| | | KD065313 | *hypothetic* | EMS63786 | 5AL, 4BL, 4DL |
| *T. aestivum* | A | 4AL_7140977 | *WD3L* | | 4AL |
| | | 4AL_7140977 | *PMEIL* | | 4AL |
| | | 5AL_2802879 | *CCCH* | | 5AL |
| | | 5AL_2802879 | *ASA1* | | 5AL |
| | | 5AL_2750251 | *HLH* | | 5AL |
| | | 5AL_2702896 | *PINX1* | | 5AL |
| | B | 4BL_6984966 | *WD3L* | | 4BL |
| | | 4BL_7040598 | *HLH* | | 4BL |
| | | 5BL_10845146 | *PMEIL* | | 5BL |
| | | 5BL_10845146 | *PMEI* | | 5BL |
| | | 5BL_10887891 | *CCCH* | | 5BL |
| | | 5BL_10887891 | *ASA1* | | 5BL |
| | D | 4DL_14359992 | *WD3L* | | 4DL |
| | | 4DL_14465353 | *HLH* | | 4DL |
| | | 5DL_4551403 | *CCCH* | | 5DL |
| | | 5DL_4551403 | *ASA1* | | 5DL |
| | | 5DL_4548681 | *PMEIL* | | 5DL |
| | | 5DL_4526272 | *PMEI* | | 5DL |

*Homologs with identity between 90% and 85% are underlined.

¶homologous to hypothetic gene coding for protein EMS63786 in *T. urartu*.

§Pseudogene due to frame shift.
