# Supplementary material for: Recurrence of Chromosome Rearrangements and Reuse of DNA Breakpoints in the Evolution of the Triticeae Genomes
Source: G3 (Bethesda). 2016 Oct 10;6(12):3837–47. doi: 10.1534/g3.116.035089 (PMC5144955; doi:10.1534/g3.116.035089)
Supplement: Supplemental Material [file supp_g3.116.035089_TableS4.pdf]

Table S2. Chromosome location of the breakpoint genes in the rye genome

Genes	Chromosome arms of CS	rye chromosome	Identities
KDM	4AL, 4BL, 4DL	7R	89% - 100%
WD3L	4AL, 4BL, 4DL	7R	90% - 100%
HLH	5AL, 4BL, 4DL	5R	82% - 89%
PINX1	5AL, 4BL, 4DL	5R	87% - 100%
FBA1	5AL, 4BL, 4DL	5R	90% - 92%
CCCH	5AL, 5BL, 5DL	5R	89% - 96%
ASA1	5AL, 5BL, 5DL	5R	89% - 98%
PMEIL	5AL, 5BL, 5DL	3R	89%
PMEI	5AL, 5BL, 5DL	5R	88%
PLC3	5AL, 5BL, 5DL	5R	91% - 92%
GAD1	5AL, 5BL, 5DL	7R	94% - 96%
