# Supplementary material for: Recurrence of Chromosome Rearrangements and Reuse of DNA Breakpoints in the Evolution of the Triticeae Genomes
Source: G3 (Bethesda). 2016 Oct 10;6(12):3837–47. doi: 10.1534/g3.116.035089 (PMC5144955; doi:10.1534/g3.116.035089)
Supplement: Supplemental Material [file supp_g3.116.035089_TableS5.pdf]

Table S3. Positions of breakpoint genes on barley chromosome arms 4HL and 5HL

Loci	Genes	Positions on 4HL	Coding strand	Chromosome arm locations in CS
MLOC_11400	*WD3L*	522,611,086 - 522,613,521	Forward strand	4AL, 4BL, 4DL
	FBA	524,198,573 - 524,199,581	Forward strand	5AL, 4BL, 4DL
	HLH	524,369,503 - 524,369,689	Reverse strand	5AL, 4BL, 4DL
MLOC_4076	*STKc*	524,372,475 - 524,375,456	Reverse strand	5AL, 4BL, 4DL
MLOC_13453	*PINX1*	525,618,209 - 525,620,120	Forward strand	5AL, 4BL, 4DL
MLOC_15830	*DAGK*	525,734,857 - 525,737,051	Forward strand	5AL, 4BL, 4DL
MLOC_57109	*KDM*	525,970,793 - 525,977,921	Forward strand	4AL, 4BL, 4DL
MLOC_57111		525,977,203 - 525,978,457	Reverse strand	4AL, 4BL, 4DL
MLOC_57112		525,979,395 - 525,980,168	Reverse strand	4AL, 4BL, 4DL

Loci	Genes	Positions on 5HL	Coding strand	Chromosome arm locations in CS
	PLC3	535,192,344 -535,193,028	Forward strand	5AL, 5BL, 5DL
MLOC_10449	*CCCH*	536,890,421 - 536,894,123	Forward strand	5AL, 5BL, 5DL
MLOC_10450	*ASA1*	536,892,909 - 536,897,697	Reverse strand	5AL, 5BL, 5DL
	PMEIL	540,673,375 -540,673,970	Reverse strand	4AL, 5BL, 5DL
