# Supplementary material for: Recurrence of Chromosome Rearrangements and Reuse of DNA Breakpoints in the Evolution of the Triticeae Genomes
Source: G3 (Bethesda). 2016 Oct 10;6(12):3837–47. doi: 10.1534/g3.116.035089 (PMC5144955; doi:10.1534/g3.116.035089)
Supplement: Supplemental Material [file supp_g3.116.035089_FigureS2.pdf]

```
NC332_5AL  TCCCGGTCGACCTGCCCGCCGTGTGCCCTGCTTTCCGTCGTGTGTTCGCTTGCTCTGACTGACGCGTGGTAGCAACAACTGTAGGAACAGA-GCAAGAGCAGAGGAGTGTCTTCCGTTAC
NC332_5DL  TCCCGGCCGACCTGCCCGCCGGGCGCTCTGCTTTCCATCGTGTGTTTGCTTGCTCTGACTGACGCGTGGTA---------------ACAGA-GCAAGAGCAGAGGAGTGTCTTCCGTTAC
NC332_4AL  TCCCGCTCCACCTGCCTGCCGGGCGCTCTGCTTTCCGTCGTGTGTTTGC-----------GACGCGTGGTAGCAAC-----TAGGAACAGA-GCAAGAGCAGAGGAGTGTCTTCTGTTAC
NC332_5BL  TCCCGGTTGACCTGCCCGCCTGGCGCTCTGCTTTCTGTGGTGTGTTCGCTTGCTCTGACTGACACGTGGTA---------------GCAGCTATAAGAGCAGAGGAGTGT--------AA
           *****    ******* ***  * ** ********  * ******* **           *** *******                ***    ****************        *

NC332_5AL  ATACACCTGACCTGCGC---------ATGTGCACAGGAGCAGCTGTCGTGACACTTTACACCAATGCTAAA-CTTTAACCTTGCTTC----CACTGCTCTGTGTTAGTGAGTGTTA----
NC332_5DL  ATACACCTGACCTGCGCTGCGCATGTATGTGCACAGGAGCAGCTCTCGTGACACTTTACACCAATGATAGA-CTTTAACCTTGCTTCTACACACTGCTCTCTGTTAGTGAGTGTTA----
NC332_4AL  ATACACCTGAACTGCGT-----------------AGGAGCAGCTCTCGTGACACTTTACACCAAATATAAA-CTTTAACCTTGCTTCTACATACTGCTCTCTTTCAGTGAGTGTTATTGT
NC332_5BL  CTACACCTGACCTGCGC-----ATGTGTGTGCACAGGAGCAGCTCTCGTGACACTTCTCACCAATGATAAAACTTTAACCTTGCTTCTACATACTGCTCTCTGCTAGCGAGTGTTA----
            ********* *****                  ********** ***********  ******   ** * ***************     ******** *   ** ********

NC332_5AL  --CCAACACAA----CACACACCAGTTTGATC-----------ATTATCCTGCATGCGCTCACGCACATCAAACATGCAGCAG-CTGATTATCTCACATGCCTACCCCA-TGCACCACTC
NC332_5DL  --CCAACACAA----CACGCACCAGCTTGATC-----------ATTATCCTGCATGCA------------------GCAGCTGATTGATTATCTCACATGCCTGCCGCA-TGCATCTCTC
NC332_4AL  TACCAACACAA----CAAGCACCAGTTTGATC-----------ATTATCCTGCATGCT-------------------CCACT--CCACGCATCCCAAACA--TGCAGCAGTAGATTTATC
NC332_5BL  --CCAACACAATTAACAAGCACCAGCTTGATGTGTGCTTGATGATTATCCTGCATGCGT-------------------------TCACGCATCCCAAACA--CGCAGCAGCTGATTTATC
             *********    **  ****** *****            **************                                 *** ** *      *  **    *    **

NC332_5AL  TAACTTGCAGATGTTACCTCGTCGGTTTG 351
NC332_5DL  TAACTTGCACATGTTACCTCTTCGGTTTG 332
NC332_4AL  TAACTTGC--------------------- 295
NC332_5BL  TC--------------------------- 301
           *
```

Figure S2. Sequence alignment of *NC332* homoeologs located in 4AL, 5AL, 5BL and 5DL chromosome arms of *T. aestivum* cv. CS.
