# Supplementary material for: Recurrence of Chromosome Rearrangements and Reuse of DNA Breakpoints in the Evolution of the Triticeae Genomes
Source: G3 (Bethesda). 2016 Oct 10;6(12):3837–47. doi: 10.1534/g3.116.035089 (PMC5144955; doi:10.1534/g3.116.035089)
Supplement: Supplemental Material [file supp_g3.116.035089_FigureS3.pdf]

```
urartu          MVKAYLRYEPALSFGVVASPESNVVYDPSGRRLLAAALDRFAAWDLKRGLPSATFTPSSSSASLAVSCVASSPAAASASASSVASGHADGSIRLWDAETG
monococcum      MVKAYLRYEPALSFGVVASPESNVVYDPSGRRLLAAALDRFAAWDLKRGLPSATFTPSSSSASLAVSCVASSPAAASASASSVASGHADGSIRLWDAETG
aestivum-4AL    MVKAYLRYEPALSFGVVASPESNVVYDPSGRRLLAAALDRFAAWDLKRGLPSATFTPSSSSASLAVSCVASSPAAASASASSVASGHADGSIRLWDAETG
                ****************************************************************************************************

urartu          ACEATLHGHRSAASAIRFAPSGAVLASGSKDCDVILWDVVAQAGLFRLRGHRDQVTDLVFLDSGKKLVTCSKDKFIRVWDLDTQHCLQIVGGHHSEIWSM
monococcum      ACEATLHGHRSAASAIRFAPSGAVLASGSKDCDVILWDVVAQAGLFRLRGHRDQVTDLVFLDSGKKLVTCSKDKFIRVWDLDTQHCLQIVGGHHSEIWSM
aestivum-4AL    ACEATLHGHRSAASAIRFAPSGAVLASGSKDCDVILWDVVAQAGLFRLRGHRDQVTDLVFLDSGKKLVTCSKDKFIRVWDLDTQHCLQIVGGHHSEIWSM
                ****************************************************************************************************

urartu          DVDSSEKFLVSGSADPELRVFRIRQSAEEGEDWNKWDALKLFGEIPRQSKERIQTIRFNKDGSLVACQVAGKTADIYRILDETEATRKAKRRLHRKKEKA
monococcum      DVDSSEKFLVSGSADPELRVFRIRQSAEEGEDWNKWDALKLFGEIPRQSKERIQTIRFNKDGSLVACQVAGKTADIYRILDETEATRKAKRRLHRKKEKA
aestivum-4AL    DVDPSEKFLVSGSADPELRVFRIRQSAEEGEDWNKWDALKLFGEIPRQSKERIQTIRFNKDGSLVACQVAGKTADIYRILDETEATRKAKRRLHRKKEKA
                ***.************************************************************************************************

urartu          SAKAAAAEGNGTVIDPLPAQDSQNPTVVVTDVFKLLQVLRTSKKICSVAFSPSNPPKGCLATLSLSLNNNVLETYSVDNEKVSKMYSVEIHGHRSDIRSL
monococcum      SAKAAAAEGNGTVIDPLPAQDSQNPTVVVTDVFKLLQVLRTSKKICSVAFSPSNPPKGCLATLSLSLNNNVLETYSVDNEKVSKMYSVEIHGHRSDIRSL
aestivum-4AL    SAKAAAAEGNGSVIDPLPAQDSQNPTVVVTDVFKLLQVLRTSKKICSVAFSPSNPPKGCLATLSLSLNNNVLETYSVDIEKVSKMYSVEIHGHRSDIRSL
                ***********:****************************************************************** *********************

urartu          ALNSEDNLLMSTSHNAVKIWNPSTGDCLRTVDSGYGLCSAFVPGNRYGLIGTKTGTLEIIDINSGNSIDVIEAHAGSIRSIVLIPDEDGTVNARGFVTGS
monococcum      ALNSEDNLLMSTSHNAVKIWNPSTGDCLRTVDSGYGLCSAFVPGNRYGLIGTKTGTLEIIDINSGNSIDVIEAHAGSIRSIVLIPDEDGTVNARGFVTGS
aestivum-4AL    ALNSEDNLLMSTSHNAVKIWNPSTGDCLRTVDSGYGLCSAFVPGNRYGLIGTKTGTLEIIDINSGNSIDVIEAHAGSIRSIVLIPDEDGTVNARGFVTGS
                ****************************************************************************************************

urartu          ADHDVKFWEYQLVQKSDSEAKYLSVTNVRTLKMNDDVLAVSIGPTGKHIAVALLDCTVKVFFLDTLKFCLSLYGHKLPVLCMDISSDGALIVTGSADKNL
monococcum      ADHDVKFWEYQLVQKSDSEAKYLSVTNVRTLKMNDDVLAVSIGPTGKHIAVALLDCTVKVFFLDTLKFCLSLYGHKLPVLCMDISSDGALIVTGSADKNL
aestivum-4AL    ADHDVKFWEYQLVQKSDSDAKYLSVTNVRTLKMNDDVLAVSIGPTGKHIAVALLDCTVKVFFLDTLKFCLSLYGHKLPVLCMDISSDGALIVTGSADKNL
                ******************:*********************************************************************************

urartu          KIWGMDFGDCHKSIFAHTDSVMDVKFVPKTHYMFSVGKDRTVKYWDADKFELLLTLEGHHAEVWCLAISSRGDFIVTGSHDRSIRRWDRTEEQLFIEEER
monococcum      KIWGMDFGDCHKSIFAHTDSVMDVKFVPKTHYMFSVGKDRTVKYWDADKFELLLTLEGHHAEVWCLAISSRGDFIVTGSHDRSIRRWDRTEEQLFIEEER
aestivum-4AL    KIWGMDFGDCHKSIFAHTDSVMDVKFVPKTHYMFSVGKDRTVKYWDADKFELLLTLEGHHAEVWCLAISSRGDFIVTGSHDRSIRRWDRTEEQLFIEEER
                ****************************************************************************************************

urartu          EKRLEETFEADLDNAVEDRYGQKDDAPDEGSVGVPGKKTKETVTAADAIIDALDTAEEEEKRLNEQKELKNDGEGTKSKPNVIMQGHSPSEYVLNAVSSV
monococcum      EKRLEETFEADLDNAVEDRYGQKDDAPDEGSVGVPGKKTKETVTAADAIIDALDTAEEEEKRLNEQKELKNDGEGTKSKPNVIMQGHSPSEYVLNAVSSV
aestivum-4AL    EKRLEETFEADLDNAVEDRYGQKDDAPDEGSVGVPGKKTKETVTAADAIIDALDTAEEEEKRLNEQKELKNDGEGTKSKPNVIMQGHSPSEYVLNAVSSV
                ****************************************************************************************************

urartu          RPNDLEQALLSLPFSDALKLMAYLKEWSLIPLKVELVCRVCLVLLQTHHNQLTTTPAARSILTALKDILYGRVKDCKDTIGFNLAAMDHIKELLTMRSDA
monococcum      RPNDLEQALLSLPFSDALKLMAYLKEWSLIPLKVELVCRVCLVLLQTHHNQLTTTPAARSILTALKDILYGRVKDCKDTIGFNLAAMDHIKELLTMRSDA
aestivum-4AL    RPNDLEQALLSLPFSDALKLMAYLKEWSLIPLKVELVCRVCLVLLQTHHNQLTTTPAARSILTALKDILYGRVKDCKDTIGFNLAAMDHIKELLTMRSDA
                ****************************************************************************************************

urartu          PFRDAKAKLLEIRQEQSKRSDRSDGGEKRKRKKPKASVQS
monococcum      PFRDAKAKLLEIRQEQSKRSDRSDGGEKRKRKKPKASVQS
aestivum-4AL    PFRDAKAKLLEIRQEQSKRSDRSDGGEKRKKKKPKASVQS
                ******************************:*********
```

Figure S3. Alignment of WD3L protein sequences of the A genomes of the genus *Triticum*.
