# Supplementary material for: Recurrence of Chromosome Rearrangements and Reuse of DNA Breakpoints in the Evolution of the Triticeae Genomes
Source: G3 (Bethesda). 2016 Oct 10;6(12):3837–47. doi: 10.1534/g3.116.035089 (PMC5144955; doi:10.1534/g3.116.035089)
Supplement: Supplemental Material [file supp_g3.116.035089_FigureS4.pdf]

```
aestivum-4AL    ATGGCGATGCATCCTCAGGTTGCTTTACTCCTCACCCTCGTCCTCCTCCTGGCGGCCGGAGATGGCGGCCTCGTCGTCGGCACTCTGCCCGCGATCATCA
urartu          ATGGCGATGCATCCTCAGGTTGCTTTACTCCTCACCCTCGTCCTCCTCCTGGCGGCCGGAGATGGCGGCCTCGTCGTCGGCACTCTGCCCGCGATCATCA
monococcum      ATGGCGATGCATCCTCAGGTTGCTTTACTCCTCACCCTCGTCCTCCTCCTGGCGGCCGGAGATGGCGGCCTCGCCGTCGGCACTCTGCCCGCGATCATCA
                ************************************************************************* **************************

aestivum-4AL    CAAGGACATGCGTCGATGTCGGCCGCGGTGGGCAAGTGGGTTACGACTCCTGCGTGGAAGCGCTCTCGGCCGACTCGGCGGCCGCGTCCGCCAAGGACGC
urartu          CAAGGACATGCGTCGATGTCGGCCGCGGTGGGCAAGTGGGTTACGACTCCTGCGTGGAAGCGCTCTCGGCCGACTCGGCGGCCGCGTCCGCCAAGGACGC
monococcum      CAAGGACATGCGTCGATGTCGGCCACGGTGGGCAAGTGGGTTACGACTCCTGCGTGGACGCGCTCTCGGCCGACCCGGCGGCCGCGTCCGCCAAGGACGC
                ************************ ********************************* *************** *************************

aestivum-4AL    ACGTGAGCTCGCCGTCGTGGCCACCAACCTTACCGTGGCCAACGTCACATCGACGGTGCTCGTCCTCGACGACCTTGTCAAGAACCTCGGGGAGTGCCTC
urartu          ACGTGAGCTCGCCGTCGTGGCCACCAACCTTACCGTGGCCAACGTCACATCGACGGTGCTCGTCCTCGACGACCTTGTCAAGAACCTCGGGGAGTGCCTC
monococcum      ACGTGAGCTCGCCGTCGTGGCCACCAACCTCGCCATGGCCAACGTCACGTCGACGGTGCTCGTCCTCGACGACCTTGTCAAGAACCTCGGGGACTGCCTC
                ******************************  ** ************* ******************************************** ******

aestivum-4AL    CGCTACTACAGGGACATGAACAAGACCCTGGAGGGCGTGGTCGGTGACCTACGTGCTGGGCGCCTCAAAGCGGCGTCTCAGAAACTCTTGGATGCCACCG
urartu          CGCTACTACAGGGACATGAACAAGACCCTGGAGGGCGTGGTCGGTGACCTACGTGCTGGGCGCCTCAAAGCGGCGTCTCAGAAACTCTTGGATGCCACCG
monococcum      CGCTACTACAGGGACATGAACAAGACCCTGGAGGGCGCGGTCGGTGACCTACGTGCTGGGCGCCTCAAAGCGGCGTCCCACAAACTGTTGGATGCCACCG
                ************************************* *************************************** ** ***** *************

aestivum-4AL    AGGCGCCCAGCTCCTGCGACATGCTCCTGTTCGAGGGGAGCGCGGAGAAGAACCCGATGAGCAAGGAGAACAACGACGCCGAGTG-------------GC
urartu          AGGCGCCCAGCTCCTGCGACATGCTCCTGTTCGAGGGGAGCGCGGAGAAGAACCCGATGAGCAAGGAGAACAACGACGCCGAGTGGCTGTCCCGAGTGGC
monococcum      AGGCGCCCAGCTCCTGCGACATGCTCTTGTTCGAGGGGAGCGCGGAGAAGAACCCGATGAGCAAGGAGAACAACGACGCCGAGTGGCTGTCCCGAATGGC
                ************************** **********************************************************             **

aestivum-4AL    ATATGCGATTGCTAGCTTGCCGGCGCCGAATTCTCGGCACTGACGTCAAGTTTAATTCTTGGTCTCATGCAACTTATTGA
urartu          ATATGCGATTGCTAGCTTGCCGGCGCCGAATTCTCGGCACTGACGTCAAGTTTAATTCTTGGTCTCATGCAACTTATTAA
monococcum      ATATGCGATTGCTAGCTTGCCGGCGCCGAATTCTCGACACTGACGTCGAGTTTAATTCTTGGTCTCATGCGGCTTATTAA
                ************************************ ********** **********************  ****** *


urartu          MAMHPQVALLLTLVLLLAAGDGGLVVGTLPAIITRTCVDVGRGGQVGYDSCVEALSADSAAASAKDARELAVVATNLTVANVTSTVLVLDDLVKNLGECL
monococcum      MAMHPQVALLLTLVLLLAAGDGGLAVGTLPAIITRTCVDVGHGGQVGYDSCVDALSADPAAASAKDARELAVVATNLAMANVTSTVLVLDDLVKNLGDCL
aestivum-4AL    MAMHPQVALLLTLVLLLAAGDGGLVVGTLPAIITRTCVDVGRGGQVGYDSCVEALSADSAAASAKDARELAVVATNLIVANVTSTVLVLDDLVKNLGECL
                ************************.****************:**********:*****.****************** :******************:**

urartu          RYYRDMNKTLEGVVGDLRAGRLKAASQKLLDATEAPSSCDMLLFEGSAEKNPMSKENNDAEWLSRVAYAIASLPAPNSRH--------
monococcum      RYYRDMNKTLEGAVGDLRAGRLKAASHKLLDATEAPSSCDMLLFEGSAEKNPMSKENNDAEWLSRMAYAIASLPAPNSRH--------
aestivum-4AL    RYYRDMNKTLEGVVGDLRAGRLKAASQKLLDATEAPSSCDMLLFEGSAEKNPMSKENNDAEWHMRLLACRRRILGTDVKFNSWSHATY
                ************.*************:***********************************  *:  .   : ..: :.
```

Figure S4. Alignment of *PMEIL* cds (top) and predicted protein sequences (bottom) from the A genomes of the genus *Triticum*. The stop codons are labeled in red.
