# Supplementary material for: Recurrence of Chromosome Rearrangements and Reuse of DNA Breakpoints in the Evolution of the Triticeae Genomes
Source: G3 (Bethesda). 2016 Oct 10;6(12):3837–47. doi: 10.1534/g3.116.035089 (PMC5144955; doi:10.1534/g3.116.035089)
Supplement: Supplemental Material [file supp_g3.116.035089_FigureS5.pdf]

```
urartu      GCTCCTGGAGATCAGGCAGGAGCAGTCGAAGCGGTCAGATAGGTCGGACGGCGGCGAGAAAAGGAAAAAGAAGAAACCCAAGGCGTCTGTACAGAGCTGA
aestivum    GCTCCTGGAGATCAGGCAGGAGCAGTCGAAGCGGTCAGATAGGTCGGACGGCGGCGAGAAAAGGAAAAAGAAGAAACCCAAGGCGTCTGTACAGAGCTGA
monococcum  GCTCCTGGAGATCAGGCAGGAGCAGTCGAAGCGGTCAGATAGGTCGGACGGCGGCGAGAAAAGGAAAAGGAAGAAACCCAAGGCGTCTGTACAGAGCTGA
            ******************************************************************** *******************************
urartu      GCAAGGATTTATTTGTCAGTTTTTACTCAACTGTTGTAGATGTACACGCCACACTGTATTATTGTGTTCATTTCATACTGTCATGAGCACAGCAGAATTT
aestivum    GCAAGGATTTATTTGTCTGTTTTTACTCAACTGTTGTAGATGTACACGCCACACTGTATTATTGTGTTCATTTCATACTGTCATGAGCACAGCAGAATTT
monococcum  GCAAGGATTTATTTGTCTTTTTTTACTCAACTGTTGTAGCTGTACACGCCACACTATATTATTGTGTTCATTTCATACTGTCATGAGCACAGCAGAATTT
            *****************  ******************** *************** ********************************************

urartu      TGAGAAGTTTGTGTGTGGGCCATTGTTACTTGGCGGTTGCCTGCTAGGGAAAGGAGGTGTACGTGTACCTCTCT--TTTAGGAAGTCAATGCTTGGTCAA
aestivum    TGAGAAGTTTGTGTGTGGGCCATTGTTACTTGGCGGTTGCCTGCTAGGGAAAGAAGGTGTACGTGTACCTCTCT--TTTAGGAAGTCAATGCTTGGTCAA
monococcum  TGAGAAGTTATTGTGTGGGCCATTGTTACTTGGCGGTTGCCTACTAGGGAAAGAAGGTGTACGTGTACCTCTCTCTTTTAGGAAGTCAATGCTTGGTCAA
            ********* ******************************** ********** ********************  ************************

urartu      ACCTTTTTTTTATGCTTTCAGAAATTGCAGCACCCCTAGCTTAAAAGTTACAAAACAAA-TTCAAGGTTTTCAGAGCATTTGAAATATCAAACTTCTGGA
aestivum    ACCTTTTTTTTATGCTTTCAGAAATTGCAGCACCCCTAGCTTAAAAGTTTCAAAACAAA-CTCAAGGTTTTCAGAGCATTTGAAATATCAAACTTTTGGA
monococcum  ACCTTTTTTT-ATGCTTTCAGAAATTGCAGCACCCCTAGCTTAAAAGTTTCAAAAAAAAATTCAAGGTTTTCAGAGCATTTGAAATATCAAACTTCTGGA
            ********** ************************************** ***** ***  ********************************** ****

urartu      AATTCGAGACCTTTTTTGAAAAGTAGGACGTTTAGAGTATAAAACTTTTGG-----GGAATTCAAA--------CT-TTTGGAGTTT-------------
aestivum    AATTCGAGACCTTTTTTGAAAAGTAGAACGTTTAGAGTATAAAACTTTTGG-----GGAATGTAAATAAAGTGCCTGTTTGGAGTCTCGCCACTCCGCTC
monococcum  AATTTGAGACCTTTTTTGAAAAGTAGAACGTTTAGAGTATAAAGCTTTTGGAGTTTGGACTTCAAA--------CT-TTTGGAGTTT-------------
            **** ********************* **************** *******     *** *  ***        ** ******** *

urartu      --GGA----------------------------------------------CTGTAAAT---------------------------------------AA
aestivum    --GGA----------------------------------------------CTGTAAAT---------------------------------------AA
monococcum  CCGGAGTGGCCAGAGCTTCAGTCGAAAATACGGAGTGGAGAAACAGATGCTCTGCAGATTCTCGTATTTTTCGGAGCGATGGATTGCCGAACAGGCTAAA
              ***                                              *** * **                                       **

urartu      AGTCTTTTACTTTTGAA-----------------------------------------------------------------------------------
aestivum    AGTCTTTTACTTTTGAA-----------------------------------------------------------------------------------
monococcum  AGTCTTTTACTTTTGAAAACCTCTAATTTTTAATAGAAAATTCTAACTTAGTACTACAAAAGAATTGAGCTGTCATTTTCTGACCAGGTGTTGTTGCAGT
            *****************

urartu      AACCTCCAATTTTTTAATAGAAAATC-------GTACTACAAAAGAATTGAGCTGTCATTTTCTGACCAGGTGTTGTTGCAGTTGCAATCCATAGGGCGA
aestivum    AACCTCCAATTTTTTAATAGAAAATC-------GTACTACAAAAGAATTGAGCTGTCATTTTCTGACCAGGTGTTGTTGCAGTTGCAATCCATAGGGCGA
monococcum  AACCTCCAATTTTTAATAGAAAATTCTAACTTAGTACTACAAAAGAATTGAGCTGTCATTTTCTGACCAGGTGTTGTTGCAGTTGCAATCCATAGGGCGA
            ***************** *******        *******************************************************************

urartu      CGCCAGATGCCACTGCTGTGATCTCAAATGCAAACCATAATTCCATATTGAGTAAACTTTTTCGGAAATGATAAATCCTGAACATTTGGAGTTTAAGCAT
aestivum    CGCCAGATGCCACTGCTGTGATCTCAAATGCAAACCATAATTCCATATTGAGTAAACTTTTTCGGAAATGATAAATCCTGAACATTTGGAGTTTAAGCAT
monococcum  CGCCAGATGCCACTGCTGTGATCTCAAATGCAAACCATAATTCCATATTGAGTAAACTTTTTCGGAAATGATAAATCCTGAACATTTGGAGTTTAAGCAT
            ****************************************************************************************************

urartu      GGCAACCCGAATCTTTTTCATGGCAATTTTTGTTCACGCGAGGTTGGCAAACATGGTAAAAACAGTTATGGATGATAGTGCTCTTATGGCATTAATGGGT
aestivum    GGCAACCCGAATCTTTTTCATGGCAGCTTTTGTTCACGCGAGGTTGGCAAACATGGTAAAAACAGTTATGGATGATAGTGCTCTTATGGCATTAATGGGT
monococcum  GGCAACCCGAACATTTTTTATGGCAACTTTTGTTCACGCGAGGTTGGCAAACATGGTAAAAACAGTTATGGATGATAGTGCTCTTATGGCATTAATGGGT
            ***********  ***** ******  *************************************************************************
```

```
urartu          CGAAACATGGTAAAGACCTG-ACTTATGTTTGATAAAATCCTTAGCATCAAACATGATAGGATTATTCAGTTTTCATCAAGTATATTCTTATAATGGTGC
aestivum        CGAAACATGGTAAAGACCTG-ACTTATGTTTGATAAAATCCTTAGCATCAAACATGATAGGATTATTCAGTTTTCATCAAGTATATCCTTATAATGGTGC
monococcum      CGAAACATGGTAAAGACCTGGACTTATGTTTGATAAAATCCTTAGCATCAAACATGATAGGATTATTCAGTTTTCATCAAGTATATTCTTATAATGGTGC
                ******************** ***************************************************************** *************

urartu          AAATGATGATGGATCAACTATTGGGCT-GACCTCATGCAC-CTCTTGCTGTTCTACGGAAGTTTGGTTTAGCATTTTGTTTTTCTCTAGAGAAGACCATG
aestivum        AAATGATGATGGATCAACTATTGGGCT-GACCTCATGCAC-CTCTTGCTGTTCTACGGAAGTTTGGTTTAGCATTTTGTTTTTCTCTAGAGAAGACCATG
monococcum      AAATGATGATGGATCAACTATTGGGCTTGACCTCATGCACTCTCTTGCTGTTCTACGGAAGTTTGGTTTAGCATTTTGTTTTTCTCTAGAGAAGACTATG
                *************************** ************ ******************************************************* ***

urartu          TAATATGATTTGGAGCTAGTTGTCTTGCGGTTATAGAACTTTGAGTTTGTAATGTCTGTTCATGCCTAATAGTCAACATATACTGTAAAAGTTTAGGATC
aestivum        TAATATGATTTGGAGCTAGTTGTCTTGCGGTTATAGAACTTTGAGTTTGTAATGTCTGTTCATGCCTAATAGTCAACATATACTGTAAAAGTTTAGGATC
monococcum      TAATATGATTTGGAGCTAGTTGTCTTGCGGTTATAGAACTTTGAGTTTGTAATGTCTGTTCATGCCTAATAGTCAACATATACTGTAAAAGTTTAGGATC
                ****************************************************************************************************

urartu          TATAGCATCCGGATACATGAGCCCATACTGGATGTGTATTTCTTCGAGGTATACTAAATTGACATGTTACCGGTTAATGCTTCATATATTATATAGGCGA
aestivum        TATAGCATCCGGATACATGAGCCCATACTGGATGTGTATTTCTTCGAGGTATACTAAATTGACATGTTACCGGTTAATGCTTCATATATTATATAGGCGA
monococcum      TATAGCATCCGGATACATGAGCCCATACTGGATGTGTATTTCTTTGAGGTATACTAAATTGACATGTTACTGGTTAATGCTTCATATATTATATAGGTGA
                ******************************************** ************************* ************************** **

urartu          TCCATTCACGTGACTTCATCCTTTGAAATCTTGCGGGATCGCGTCCTCATATAGAGTATAATAGTACTCTTCTTTGGAGCTGAATCCATGTAGCATGAAG
aestivum        TCCATTCACGTGACTTCATCCTTTGAAATCTTGCGGGATCGCGTCCTCATATAGAGTATAATAGTACTCTTCTTTGGAGCTGAATCCATGTAGCATGAAG
monococcum      TCCATTCACGTGACTTCATCCTTTGAAATCTTACGGGATCGCGCCCTCATATAGAGTATAATAGTACTCTTCTTTGGAGCTGAATCCATGTAGCATGAAG
                ******************************** ********** *********************************************************

urartu          TTTTGCTCACACGGTA-CTACTTAATGGACTTCGGTTTGCCACAAAATGAATTAACTGACCTTGGTTGTGGCTGACTTGGAACGATGTGTTTGAACATTG
aestivum        TTTTGCTCACACGGTAACTACTTAATGGACTTCGGTTTGCCACAAAATGAATTAACTGACCTTGGTTGTGGCTGACTTGGAACGATGTGTTTGAACATTG
monococcum      TTTTGCTCACACGGTAACTACTTAATGGACTTCGGTTTGCCACAAAATGAATTAACTGACCTTGGTTGTGGCTGGCTTGGAACGATGTGTTTGAACATTG
                **************** ********************************************************* *************************

urartu          CACCTTCAGTCCTTTGCACACTCTTGTTATTTGTTTAAGAAGTTCCTCATGTTTATTTTGTTATGTGTATAGCTTTCAGCCTGCCACTGTTCAAATCTGC
aestivum        CACCTTCAGTCCTTTGCACACTCTTGTTATTTGTTTAAGAAGTTCCTCATGTTTATTTTGTTATGTGTATAGCTTTCAGCCTGCCACTGTTCAAATCTGC
monococcum      CACCTTCAGTCCTTTGCACACTCTTGTTATTTGTTTAAGAAGTTCCTCATGTTTATTTTGTTATGTGTATAGCTTTCAGCCTGCCACTGTTCAAATCTGC
                ****************************************************************************************************

urartu          AAGTGCCTCATGCCATATGCTACTATGGCGATTGTTGAGCTATGTAGTCGCCAGCACTGCACGAGTTCAGAAATGAAGTATTCTGGTTTGACCATCTGAG
aestivum        AAGTGCCTCACGCCATATGCTACTATGGCGATTGTTGAGCTATGTAGTTGCCAGCACTGCACGAGTTCAGAAATGAAGTATTCTGGTTTGACCATCTGAG
monococcum      AAGTGCCTCACGCCATATGCTACTATGGCGATTGTTGAGCTATGTAGTTGCCAGCACTGCACAAGTTCAGAAATGAAGTATTCTGGTTTGACCATATGAG
                ********** ************************************* ************* ******************************** ****

urartu          TTCTACATTATGTTGTGTTGTGAGACCCAACACTTATTTTGGGGACAGGAAAGCAAGCATGGACGGCATACACAGAGTCTATTCTGAACATGTCTACAGT
aestivum        TTCTACATTATGTTGTGTTGTGAGACCCAACACTTATTTTGGGGACAGGACAGCAAGCATGGACGGCATACACAGAGTCTATTCTGAACATGTCTACAGT
monococcum      TTCTACATTATGTTGTGTTGTGAGACCCAACACTTACTTTGGGGACAGGACAGCAAGCATGGACGGCATACACAGAGTCTATTCTGAACATGTCTACAGT
                ************************************ ************* *************************************************

urartu          AAGATCATGGACATGTGTATGGATTATGAAAAATATATTActatctccgtcacggtttagaaggcgcggttgaaaattctctggaacctaggtggttatc
aestivum        AAGATCGTGGACATGTGTATGGATTATGAAAAATATATTActacctc-gtc-cggtttagaaggcgcggttgaaaattctctgggacctaggtgattatc
monococcum      GAGATCGTGGACATGTGTATGGATTATGAAAAATATATTA------------------------------------------------------------
                 ***** *********************************
```

```
urartu       tattggttatgagatgggctaaaaaatagcattcacactacgcatgcatataaagatgggctaaaaaatagcattcacactacgcatgcatatagaaata
aestivum     tattggttatgagatgggctaaaa------attcacactacgcatgcatataaagatgggctaaaaaatagcattcacactacgcatgcatatagaaata
monococcum   ----------------------------------------------------------------------------------------------------

urartu       gtactaattagctattagaaataaatgcaatgcgccctaaattttgtctattgtgaaaatgcacgcaactttaactgtgccttctaaaccgtgacggagg
aestivum     gtactaattagctactagaaataaatgcaatgcgccctaaactttgtctattgtggaaacgcacgcaaatttaactatgccttctaaaccgtgacggagg
monococcum   ----------------------------------------------------------------------------------------------------

urartu       gagtaTATGTTTCGAATTTTACCACAGGAAATTTATGGCTGCAGATGCAGTTGTGCAATGGAGTGGGGGTGGTGGAGGAGAGGGGTGGATAGTTGGAGCA
aestivum     aagtaTATGTTTCGAATTTTACCACAGAAAATTTATGGCTGCAGATGCAGTTGTGCAATGGAGTGGGGGTGGTGGAGGAGAGGGGTGGATAGTTGGAGCA
monococcum   -----TGTGTTTCGAATTTTACCATAGAAAATTTATGGCTGCATATACAGTTGTGCAATGGAATGGGGGTGGTGGAGGAGAGGGGTGGATAGTTGGAGCA
                  * ***************** ** *************** ** *************** *************************************

urartu       ATGGAGAATGAAATGGTGCATTTAATTTTATAGTTACTATCCTCACAACTTATTTTATATAGGCAATAGTGAGTGAAGCGATGGCAGATCATAGATAAAG
aestivum     ATGGAGAATGAAATGGTGCATTTAATTTTATAGTTACTATCCTCACAACTTATTTTATATAGGCAATAGTGAGCGAAGCGATGGCAGATCATAGATAAAG
monococcum   ATGGAGAATGAAATGGTGCATTTAATTTTATAGTTACTATCCTCACAACTTATTTTATATAGGCAACACTGAGCGAAGCGATGGCACATCATAGATAAAG
             ****************************************************************** * **** ************ *************

urartu       TTGCATAGAAAAGTAGTACTTGTCGACAATAATTATAGGTTGCCTGCCGAGTCTTGTCAAGATTAACCTTCATTTAACATATTGTATTATATGATGTGAA
aestivum     TTGCATAAAAAAGTAGTACTTGTCGACAATAATTATAAGTTGCCTGCCGAGCCTTGTCAAGATTAACCTTCATTTAACATATTGTATTATATGATGTGAA
monococcum   TTGCAGAAAAAAGTAGTACTTGTCGACAATAATTATAGGTTGCCTGCCGAGTCTTGTCAAGATTAACCTTCATTTAACATGTTGTATTATATGATGTGAA
             ***** * ***************************** ************* **************************** *******************

urartu       AATGATGATGTAGAATTTGCAGTTAAAGCAATTTTGCAATAGTTGTGGTTCTGTAAATGAAAATATACTGTCGGCGTCGTGCGTGGGTGCCAGCAGTCCT
aestivum     AATGATGATGTAGAATTTGCAGTTAAAACAATTTTGCAATAGTTGTGGTTCTGTAAATGAAAATATACTGTCGGCGTCGTGCGTGGGTGCCAGCAGCCCT
monococcum   AATGATGATGTAGAATTTGCAGTTAAAACAATTTTGCAATAGTTGTGGTTCTGTAAATGAAAATATACGGTCGGCGTCGTGCGTGGGTGCCAGCAGCCCT
             *************************** **************************************** *************************** ***

urartu       GAACTGGCGCAGAGAG--GGCCGAACAGGAGGTCCCCACTCATGCAGCCTGTCTTGTCTGGAGACAAAATGTGGATCAACAACAATTCCCGTATGAGATG
aestivum     GAACTGGCGCAGAGAG--GGCCGAACAGGAGGTCCCCACTCATGCAGCCTGTCTTGTCTGGAGACAAAATGTGGATCAACAACAATTCCCGTATGAGATG
monococcum   AAACCGGCGCAGAGAGAGGGCTGAACAGGAGGTCCCCACTCATGCAGCCTGTCTTGCCTGGAGACAAAATGTGAACCAGCAACAATTCCCGTATGAGATG
              *** ***********  *** ********************************** **************** * ** *********************

urartu       TTATCTGTCACATTAGAGCCGTTCTGTATATGGAATTGACATCACACACGCTGGACATCAAATGGCAATTTCATTTTATGCGATGGACCGAGTAAATGGC
aestivum     TTATCTGTCACATTAGAGCCGTTCTGTATATGGAATTGACATCACACACGCTGGACATCAAATGGCAATTTCATTTTATGCGATGGACCGAGTAAATGGC
monococcum   TTATCTGTCACATTAGAGCCGTTCTGTATATGGAATTGACATCTCACACGTTGGACATCAAATGGCAATTTCATTTTATGCGATGGACCAAGCAAATGGC
             ******************************************* ****** ************************************** ** *******

urartu       AATCACATCAACCACCAGCTAGCTGCGTAGCTAGATTAATAAGTTGCATGAGACCAAGAATTAAACTTGACGTCAGTGCCGAGAATTCGGCGCCGGCAAG
aestivum     AATCACATCAACCACCAGCTAGCTGCGTAGCTAGATCAATAAGTTGCATGAGACCAAGAATTAAACTTGACGTCAGTGCCGAGAATTCGGCGCCGGCAAG
monococcum   AATCACATCAACCACCAGCTAGCTGCGTAGCTAGATTAATAAGCCGCATGAGACCAAGAATTAAACTCGACGTCAGTGTCGAGAATTCGGCGCCGGCAAG
             ************************************ ******  ********************** ********************************

urartu       CTAGCAATCGCATATGCCACTCGGGACAGCCACTCGGCGTCGTTGTTCTCCTTGCTCATCGGGTTCTTCTCCGCGCTCCCCTCGAACAGGAGCATGTCGC
aestivum     CTAGCAATCGCATATGC-------------CACTCGGCGTCGTTGTTCTCCTTGCTCATCGGGTTCTTCTCCGCGCTCCCCTCGAACAGGAGCATGTCGC
monococcum   CTAGCAATCGCATATGCCATTCGGGACAGCCACTCGGCGTCGTTGTTCTCCTTGCTCATCGGGTTCTTCTCCGCGCTCCCCTCGAACAAGAGCATGTCGC
             *****************             ********************************************************** ***********
```

**Figure S5.** Sequence alignment of the 4AL junctions of the 4AL/5AL translocation from *T. monococcum*, *T. urartu* and *T. aestivum*. The 5' end is centromere-bound and connected to the 3' part of *WD3L* and 3' end is telomere-bound and connected with 3' part of *PMEIL*. The 3' UTRs, stop codons and coding regions are highlighted in blue, red and green, respected. Poly(dA:dT) tracts (>6 bp) are highlighted in grey, MITE DTT_Plutto is highlighted in lower case, and the 41-bp tandem duplications within the MITE in *T. urartu* and *T. aestivum* and 83-bp duplication in *T. monococcum* are underlined. The binding sites of primers for PCR amplification of 4AL junction (Fig. 4) are indicated in red bold. The distance between the 3' UTR ends of *WD3L* and PMEIL is 2,155 bp in *T. urartu*, 2,150 bp in *T. aestivum* and 2,100 bp in *T. monococcum*.
