# Supplementary material for: Recurrence of Chromosome Rearrangements and Reuse of DNA Breakpoints in the Evolution of the Triticeae Genomes
Source: G3 (Bethesda). 2016 Oct 10;6(12):3837–47. doi: 10.1534/g3.116.035089 (PMC5144955; doi:10.1534/g3.116.035089)
Supplement: Supplemental Material [file supp_g3.116.035089_FigureS6.pdf]

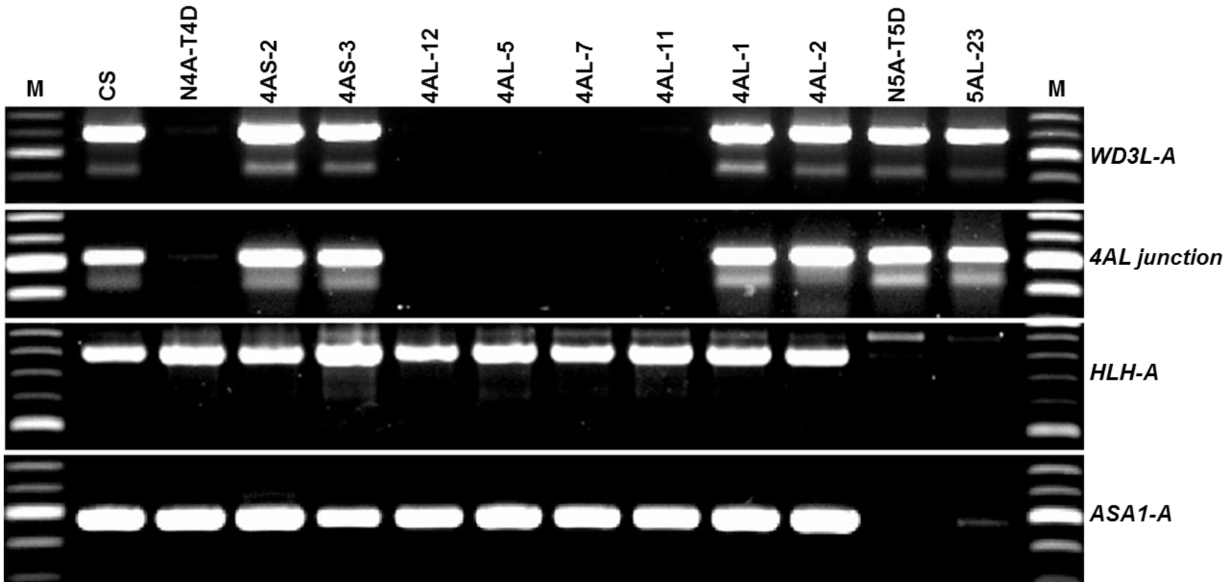

Figure S6. Deletion mapping of breakpoint genes by PCR using the A genome-specific primers. The CS genetic stocks are indicated on the top, and the genes and 4AL junction are indicated in the right of the pictures. The CS genetic stocks are described in Table S3. The sequence of primers are listed in Table S4. M: 100-bp ladders. The bright bands indicate 500 bp.
