# Supplementary material for: Recurrence of Chromosome Rearrangements and Reuse of DNA Breakpoints in the Evolution of the Triticeae Genomes
Source: G3 (Bethesda). 2016 Oct 10;6(12):3837–47. doi: 10.1534/g3.116.035089 (PMC5144955; doi:10.1534/g3.116.035089)
Supplement: Supplemental Material [file supp_g3.116.035089_FigureS7.pdf]

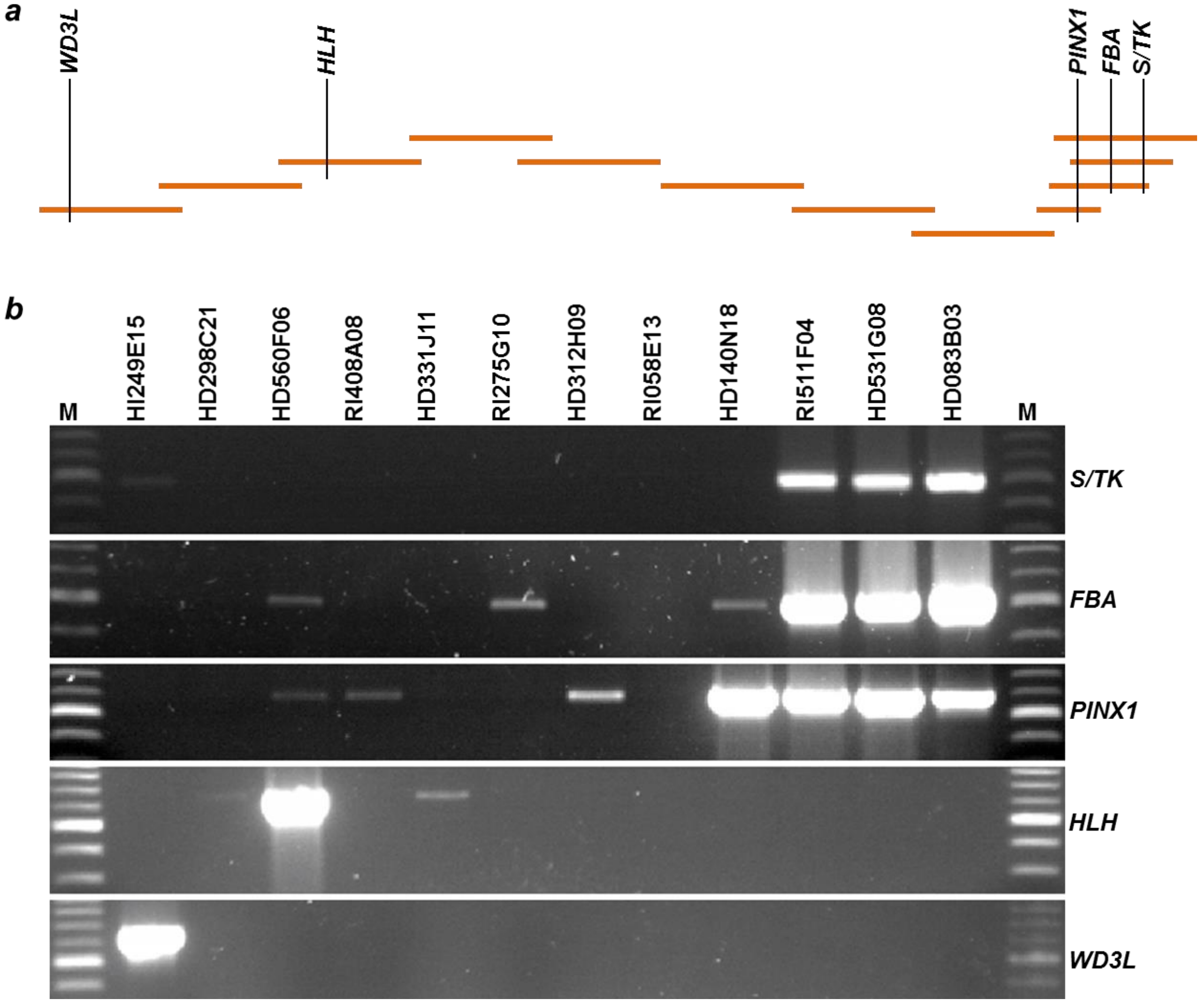

Figure S7. Determination of the gene order in BAC contigs ctg139 by PCR assays. (***a***) A diagram of the terminal portion of ctg139 anchored by markers BE639039, AT4D4118 and AT4D4119. The horizontal bars represent the overlapping BACs in the contigs, and the vertical bars indicate the positions of the markers and positions of genes deduced from PCR assay. The left end is toward the centromere, and right end toward the telomere of 4DL chromosome arm. (***b***) PCR assay of BACs for localizing the genes. BAC clones are indicated on the top of the figure in the same order as they are in ctg139, and the genes assayed are indicated in the right of the figure. M: 100-bp ladder. The bright bands indicate 500 bp.
