# Supplementary material for: Recurrence of Chromosome Rearrangements and Reuse of DNA Breakpoints in the Evolution of the Triticeae Genomes
Source: G3 (Bethesda). 2016 Oct 10;6(12):3837–47. doi: 10.1534/g3.116.035089 (PMC5144955; doi:10.1534/g3.116.035089)
Supplement: Supplemental Material [file supp_g3.116.035089_FigureS8.pdf]

```
ASA-A1   MGHP--TPSRGHLPHRSSLAVAAR------------------------------------------QLEHRCSMACPLLCSKKMDPTKKI 46
ASA-B1   MAAAGLALSLSLRPAQPSARVPRR------------------------------------------ALPPPLPPPQGRLAVGCRASALAS 48
ASA-D1   MKRLLQIIEASEPMHRPRIAVIKPPQSFVWNDGYGNDDIDKFMFNEFITSLDSDSEDAEMMMSIEEEFEKKCTTALKMLAYGKEADSIDA 84
         *       . .    :.   *                                              :    . .   *.      :

ASA-A1   NPAS-----------PWSLSPLQLCREATAVVSCIN------------------AVREEEEAFREAAAAGHTLLPLQRCIFSDHLTPVLA 107
ASA-B1   PPLS-----------PLAR-PGVACRAATAFQKLDAV-----------------AVREEEEAFRSGAAAGHTLLPLQRCIFSDHLTPVLA 109
ASA-D1   EIQMGETMVLNTTKCTTALKMLAYGKEADSIDAEIQMGETMVLNTTVQCPHCGDAVREEEEAFRSGAAAGHTLLPLQRCIFSDHLTPVLA 180
                        . :       : * : .                      **********..************************

ASA-A1   YRCLVREDDREAPSFLFESVEQASEGTNVGRYSVVGAQPAMEIVAKANQVTVMDHEMRTKEEQYAADPMTVPRDIMEQWNPQITLDGLPD 197
ASA-B1   YRCLVREDDREAPSFLFESVEQASEGTNVGRYSVVGAQPAMEIVAKANQVTVMDHEMRTKEEQYAADPMTVPRDIMAQWNPQITVDGLPD 199
ASA-D1   YRCLVREDDREAPSFLFESVEQASEGTNVGRYSVVGAQPAMEIVAKANQVTVMDHEMRTKEEQYAADPMTVPRDIMEKWNPQITVDGLPD 270
         **************************************************************************** :******:*****

ASA-A1   AFCGGWVGFFSYDTVRYVETKKLPFSKAPEDDRNLPDIHLGLYNDVVVFDHVEKKTHVIHWVRLDCYHSIDEAYEDGKNRLEALLSRLHS 287
ASA-B1   AFCGGWVGFFSYDTVRYVETKKLPFSKAPEDDRNLPDIHLGLYNDVVVFDHVEKKTHVIHWVRLDCYHSIDEAYEDGKNRLEALLSRLHS 289
ASA-D1   AFCGGWVGFFSYDTVRYVETKKLPFSKAPEDDRNLPDIHLGLYNDVVVFDHVEKKTHVIHWVRLDCYNSIHKAYEDGKNRLEALLSRLHS 360
         *******************************************************************:**.:******************

ASA-A1   SNVPTLSAGSIKLNVGQFGSALQKSTMSSEDYKKSVVQAKEHILAGDIFQVVLSQRFERRTFADPFEVYRALRIVNPSPYMAYLQARGCI 377
ASA-B1   SNVPTLSAGSIKLNVGQFGSALQKSTMSSEDYKKSVVQAKEHILAGDIFQVVLSQRFERRTFADPFEVYRALRIVNPSPYMAYLQARGCI 379
ASA-D1   SNVPTLSAGSIKLNVGQFGSALQKSTMSSKDYKKSVVQAKEHILAGDIFQVVLSQRFERRTFADPFEVYRALRIVNPSPYMAYLQARGCI 450
         *****************************:************************************************************

ASA-A1   LVASSPEILTRVAKRTVVNRPLAGTIRRGKTKAEDKVLEQLLLSDEKQRAEHIMLVDLGRNDVGKVSKPGTVKVEKLMNIERYSHVMHIS 467
ASA-B1   LVASSPEILTRVAKRTVVNRPLAGTIRRGKTKDEDKVLEQLLLSDEKQRAEHIMLVDLGRNDVGKVSKPGTVKVEKLMNIERYSHVMHIS 469
ASA-D1   LVASSPEILTRVAKRTVVNRPLAGTIRRGKTKAEDKVLEQLLLSDEKQRAEHIMLVDLGRNDVGKVSKPGTVKVEKLMNIERYSHVMHIS 540
         ******************************** *********************************************************

ASA-A1   STVTGELCDDLTCWDALRAALPVGTVSGAPKVRAMELIDEMEVTMRGPYSGGFGQISFHGDMDIALALRTIVFPTASRFDTMYSYAADSS 557
ASA-B1   STVTGELRDELTCWDALRAALPVGTVSGAPKVRAMELIDEMEVTMRGPYSGGFGQISFRGDMDIALALRTIVFPTASRFDTMYSYGTDSS 559
ASA-D1   STVTGELRDELTCWDALRAALPVGTVSGAPKVRAMELIDEMEVTMRGPYSGGFGQISFRGDMDIALALRTIVFPTASRFDTMYSYATDSS 630
         ******* *:************************************************:**************************.:***

ASA-A1   NARQEWVAHLQTGAGIVADSKPDDEQQECQNKAAGLARAIDLAESTFLDFSGM 610
ASA-B1   NARQEWVAHIQTGAGIVADSKPDDEQQECQNKAAGLARAIDLAESTFLDLSDA 612
ASA-D1   NARQEWVAHLQTGAGIVADSKPDDEQQECQNKAAGLARAIDLAESTFVDFSDA 683
         *********:*************************************:*:*.
```

Figure S8. Sequence alignment of proteins ASA-A1, ASA-B1 and ASA-D1 from the *ASA1* homeologous genes on 5AL of *T. urartu*, 5BL of *T. aestivum* cv. Chinese Spring and 5DL of *A. tauschii*, respectively. The anth_synt_I_N domain is highlighted in light blue, and the chorismate_bind domain is highlighted in yellow.
