# Supplementary material for: Recurrence of Chromosome Rearrangements and Reuse of DNA Breakpoints in the Evolution of the Triticeae Genomes
Source: G3 (Bethesda). 2016 Oct 10;6(12):3837–47. doi: 10.1534/g3.116.035089 (PMC5144955; doi:10.1534/g3.116.035089)
Supplement: Supplemental Material [file supp_g3.116.035089_FigureS9.pdf]

```
aestivum        AATCATTTTCAAAGGTTGGGACTTCGGTCTCATTTACCTGCGAGGCAAATTTTTTT-GATCCAAAAGCTAAGAAAAAAGAAGAAAACCAAGAAGCCAAAT
urartu          AATCATTTTCAAAGGTTGGGACTTCGGTCTCATTTACCTGCGAGGTAAATTTTTTTTGATCCAAAAGCTAAGAAAAAGGAAGAAAACCAAGAAGCCAAAT
monococcum      AATCATTTTCAAAGGTTGGGATTTCGGTCTCATTTACCTGTGAGGCAAAAATTTTT--ATCCAAAAGCTAAGAAAAAGGAAGAAAACCAAGAAGCCAAAT
                ********************* ****************** **** ***  *****  ****************** ***********************

aestivum        AAACAAAAAAA-CATCTAAAAACCGAAAATGCGTACAGAAGAAAAATCCAAAGGAAGCGCCCATAATGCGACGGATAACGTGCTCCCAATCCACCCCAAA
urartu          AAACAAAAAAA-CATCTAAAA-CCGAAAATGCGTACAGAAGAAAAATCCAAAGGGAGCGCCCAGAATGCGACAGATAACGTGCTCCCAATCCACCCCAAA
monococcum      AAACAAAAAAAACATCTAAAAACCAAAAATGCGTATAGAAGAAAAATCCAAAGAAAGCGCCTAAAACGCGACAGATAACGTGCTCCCAATCCACCCCAAA
                *********** ********* ** ********** *****************  ****** * ** ***** ***************************

aestivum        TGACCCATGAGAGGCTTTTTTAATTGTTTGAGTTGCTTATCACATATGTCACGTGATAACCCTTGGGAAGGCTTTCGAAGCAGTACTCCTTAATTAGTTT
urartu          TAACTCATGAGAGACTTTTTTAATTGTTTGAGTTGCTTATCACATATGTCACGTGATGATCCTTGGGAAGGCTTTCGAAGCAGTACTCCTTAATTAGTTT
monococcum      TGACCCATGAGAGGCTTTTTTAATTGTTTGAGTTGCTTATCACATATGTCACGTGATGACCCTTGGGAAGGCTTTCGAAGCAGTACTCCTTAATTAGTTT
                * ** ******** ******************************************* * ****************************************

aestivum        CTCCTGTCATGCAGACGTACTAGATTAGCTGCTATTAGACCGCTATATGTGCGCTAATGGCCGGAAGATTTAACCTGTGTGCACCCAACTTAGGTAGAAG
urartu          CTCCTGTCATGCAGACGTACTAGATTAGCTGCTATTAGACCGCTATATGTGCGCTAATGGCCGGAAGATTTAACCTGTGTGCACCCAACTTAGGTAGAAG
monococcum      CTCCTGTCATGCAGACGTACTAGATTAGCTGCTATTAGACCGCTATATGTGCGCTAAAGGCCGGAAGATTTAACCTGTGTGCACCCAACTTAGGTAGAAG
                ********************************************************* ******************************************

aestivum        CCCAACAGCCCATCGGCTGAGAGTTATCATCACGGCCTGGAGTTGTTTTTCTGTCCTCAGCCATACAGTTAAACGATTGCGAGCCGCTCAAAAAAAAAAA
urartu          CCCAACAGCCCATCGGCTGAGAGTTATCATCACGGCCTGGAGTTGTTTTTCTGTCCTCAGCCATACAGTTAAACGATTGCGAGCCGCTCAAAAAAAAAA-
monococcum      CCCAACAGCCCATCGGCTGAGAGTTATCATCACGGCCTGGAGTTGTTTTTCTGTCCTCAGCCATACAGTTAAACGATTGCGAGCGGCTCAAAAAAAAAA-
                ************************************************************************************ ************** 

aestivum        GTTAAATGATTGTGATGACCAAGAAAATTAATGGCATTCACGATCCAGCAGTTCTAAAGGCGATCGCCTGCAGGAGGCACTGTCATTAGCAGAAGACTTG
urartu          GTTAAACGATTGCGATGACCAAGAAAATTAATGGCATTCACGATCCAGCAGTTCTAAAGGCGATCGCCTGCAGGAGGCACTGTCATTAGCAGAAGACTTG
monococcum      GTTAAACGATTGCAATGACCAAGAAAATTAATGGCATTCACGATCCAGCAGTTCTAAAGGCGATCGCCTGCAGGAGGCACTGTCATTAGCAGAAGACTTG
                ****** *****  **************************************************************************************

aestivum        CTTCTACAGAACTTTGTGGTGATGTTTGATTCAAAATAAGTGATTGGTGATATCCACAACGACAATCAAAGGTAATATGGCACTATTGTTAGTGAAATTA
urartu          CTTCTACAGAACTTTGTGGTGACATTTGATTCAAAACAAGTGATTAGTGATATCCACAACGACAATCGAAGGTAATATGGCACTATTGTTAGTGAAATTA
monococcum      CTTCTACAGAACTTTGTGGTGACGTTTGATTTAAAACAAGTGATTGGTGATATCCACAACGACAATCAAAGGTAATATGGCACTATTGTTAGTGAAATTA
                **********************  ******* **** ******** ********************* ********************************

aestivum        CTCATAGAATTACCATGTTTAATTGTAATTTTATTTTCGAAAGTATAGAGTCGAATAGAGATGCTCATAGTCTAGCCAAGTTTGCACATTCTTTAGATCA
urartu          CTCATAGAATTACCATGTTTAATTGTAATTTTATTTTTGAAAGTATAGAGTCGAATAGAGATGCTCATAGTCTAGCTAAGTTTGCACATTCTTTAGGTCA
monococcum      CTCATAGAATTACCATGTTTAATTGTAATTTTATTTTCGAAAGTATAGAGTCGAATAGAGATGCTCATAGTCTAGCCAAGTTTGCACATTCTTTAGGTCA
                ************************************* ************************************** ******************* ***

aestivum        AGGGCGCCACTTGTGGCTTGGTCAGCCCATGATCCCAGATGTATTTCACTATCTCTGGGTTTTGATCAATAAACTTGGCTCTATGCCTAAAAAAGATTGA
urartu          AGGGCGCCACTTGTGGCTTGGTCAGCCCATGATCCCAGATGTATTTCACTATCTCTGGGTTTTGATCAATAAACTTGGTTCTATGCCTAAAAAAGATTGA
monococcum      AAGGCGCCACTTGTGGCTTGGTCAGCCCATGATCCCAGATGTATTTCACTATCTCTGGGTTTTGATCAATAAACTTGGTTCTACGCCTAAAAAAGATTGA
                * **************************************************************************** **** ****************

aestivum        GTCCCCTAAAAAAAGGTTAGCCGATTGGAGAACTAGATATATCGATTAGTTATCGAACCATGTGTCTCAGCGCCGCTGCTAAGGCTACCGCTGTCGTCAC
urartu          GTCCCCTAAAAAAAGGTTAGCCGATTGGAGAACTAGATATATCAATTAGTTATCGAACCATGTGTCTCAGCGCCGCTGCTAAGGCTACCGCTGTCGTCAC
monococcum      GTCCCCTAAAAAAAGGTTAGCCGATTGGAGAACTAGATATATCGATTAGTTATCGAACCATGTGTCTCAGCGCCGCTGCTAAGGCTACCGCTGTCGTCAC
                ******************************************* ********************************************************
```

```
aestivum      TCGCAGTCGTTCAGGGAGGCAAGGAGATCAGAGCAGGATATGACCAAGTGCTAGCCACTCCAGGGCTCTCTCAGCGACGCTGGTGTATCATCGTGTCGAA
urartu        TCGCCGTCGTTCAGGGAGGCAAGGAGATCAGAGCAGGATATGACCAAGTGCTAGCCACTCCAGGGCTCTCTCAGCGACGCTGGTGTATCATCGTGTCGAA
monococcum    TCGCCGTCGTTCAGGGAGGCAAGGAGATCAGAGCAGGATATGACCAAGTGCTAGCCACTCCGGGGCTCTCTCAGCGACGCTGGTGTATCATCGTGTCGAA
              **** ******************************************************** **************************************

aestivum      ATATGTCGGACGTACCGTTGAAGTGTTGATCACTGTAAGTATTTTATAAGATGGTCACCTCGTGTATACACCAGCTCTGGTAGATACTAGCTAGTTACCA
urartu        ATATGTCGGACGTACCGTTGAAGTGTTGATCACTGTAAGTATTTTATAAGATGGTCACCTCGTGTATACACCAGCTCTGGTAGATACTAGCTAGTTACCA
monococcum    ATATGTCGGACATACCGTTGAAGTGTTGATCACTGTAAGTATTTTATAAGATGGTCACCTCGTGTATACACCAGCTCTGGTAGATACTAGCTAGTTACCA
              *********** ****************************************************************************************

aestivum      GTTTTTCTTTGATGAGTTGACAAATTTCCACCAACCAATCTTGTTTAACTATAACATACTTAAATATGAAACAACATAATAGTTTATCAATTTGATTAGT
urartu        GTTTTTCTTTGATGAGTTGACAAATTTCCACCAACCAATCTTGTTTAACTATAACATACTTAAATATGAAACAACATAATAGTTTATCAATTTGATTAGT
monococcum    GTTTTTCTTTGATGAGTTGACAAATTTCCACCAACCAATCTTGTTTAACTATAACATACTTAAATATGAAACAACATAATAGTTTATCAATTTGATTAGT
              ****************************************************************************************************

aestivum      TTTTCTTTCAATTTTATTATGCCTCAATTATAAAGAGG---------------------------AAAACAACAACGGTGTGAAACTCTTTTTACAAGCC
urartu        TTTTCTTTCAATTTTATTATGCCTCAATTATAAAGAGGCTTTCGCAAAAAAAAATTATAGAGAGGAAAACAACAACGGTGTGAAACTCTTTTTACAAGCC
monococcum    TTTTCTTTCAATTTTATTATGCCTCAATTATAAAGAGG---------------------------AAAACAACAACGGTGTGAAACTCTTTTTACAAGCC
              **************************************                           ***********************************

aestivum      AATTGCTTCCACCTCCAACACTCTTAGTGTTGAGACTATCAACTTTGCTTGACACGTGTGATAGGTTGTGGCTAAAACCTATCTAAAAAGTTAGATAATT
urartu        AATTGCTTCCACCTCCAACACTCTTAGTGTTGAGACTATCAACTTTGCTTGACACGTGTGGTAGGTTGTGGCTAAAACCTATCTAAAAAGTTAGATAATT
monococcum    AATTGCTTCCACCTCCAACACTCTTAGTGTTGAGACTATCAACTTTGCTTGACACGTGTGGTAGGTTGTGGCTAAAACCTATCTAAAAAGTTAGATAATT
              ************************************************************ ***************************************

aestivum      AATCTTCACTCTAAAAATATAATCTTATGGGAATGTAGCCTTGTCGTTGCGAGGCATATGTTGCCATATTTTGATATCAATGCCATATTGAGTTGAAAGT
urartu        AATCTTCACTCTAAAAATATAATCTTATGGGAATGTAGCCTTGTCGTTGCGAGGCATATGTTGCCATATTTTGATATCAATGCCATATTGAGTTGAAAGT
monococcum    AATCTTCACTCTAAAAATATAATCTTATGGGAATGTAGCCTTGTCGTTGCGAGGCATATGTTGCCATATTTTGATATCAATGCCATATTGAGTTGAAAGT
              ****************************************************************************************************

aestivum      CCTAATATATTGTGACATTTTATGAGGGCTATGGTTATGTGCATCACTCGATGTAGAGACTCAAGACCTCCCTCTTTTTGAAGAAAAAAATGTGTAATGA
urartu        CCTAATATATTGTGACATTTTATGAGGGCTATGGTTATGTGCATCACTCGATGTAGAGACTCAAGACCTCCCTCTTTTTGAAGAAAAAAATGTGTAATGA
monococcum    CCTAATATATTGTGACATTTTATGAGGGCTATGGTTATGTGCATCACTCGATGTAGAGACTCAAGACCTCCCTCTTTTTGAAGAAAAAAATGTGTAATGA
              ****************************************************************************************************

aestivum      TCATTGTCTTTCTCGCCCCC-TCATGCCTACATCCTGTCTCCGCCCTGCGGCTTCTGCATATTTGGTTCACATACAACTGGTAACCAGATGATTCCCCGC
urartu        TCATTGTCTTTCTCGCCCCC-TCATGCCTACATCCTGTCTCCGCCCTGCGGCTTCTGCATATTTGGTTCACATACAACTGGTAACCAGATGATTCCCCGC
monococcum    TCATTGTCTTTCTCGCCCCCCTCATGCCTATATCCTGTCTCCGCCCTGCGGCTTCTGCATATTTGGTTCACATACAACTGGTAACCAGATGATTCCCCGC
              ******************** ********* *********************************************************************

aestivum      GCGTTGATGTGCGAAATATTGCCTAAAGTTGGTGTGTTTTACTGATCAAACCGACTCTCCTAAAAGGATTAACAAAATGATGCATAAATAATGTTGCTTT
urartu        GCGTTGATGTGTGAAATATTGCCTAAAGTTGGTGTGTTTTACTGATCAAACCGACTCTCCTAAAAGGATTAACAAAATGATGCATAAATAATGTTGCTTT
monococcum    GCGTTGATGTGCGAAATATTGCCTAAAGTTGGTGTGTTTTACTGATCAAACCGACTCTCCTAAAAGGATTAACAAAATGATGCATAAATAATGTTGCTTT
              *********** ****************************************************************************************

aestivum      GATAATGATACTTTAGAATGGTACATGGCTAATAATACATACGTAAGATCAATCATTATACTAATATCATTAAAAGTTTAGACAACAAATGAACGATCGG
urartu        GATAATGATACTTTAGAATGGTACATGGCTAATAATACATACGTAAGATCAATCATTATACTAATATCATTAAAAGTTTAGACAACAAATGAACGATCGG
monococcum    GATAATGATACTTTAGAATGGTACATGGCTAATAATACATACGTAAGATCAATCATTATACTAATATCACTAAAAGTTTAGACAACAAATGAACGATCGG
              ********************************************************************* ******************************
```

```
aestivum      AACAGAGGATATTATGGCCAATTAACCATGGAACAAATTGGTTAAAGCTTTTCAATATATGCTACTATCTTTGCTTCTTTGCAAGTGGGAAAACTATCAA
urartu        AACAGAGGATATTATGGCCAATTAACCATGGAACAAATTGGTTAAAGCTTTTCAATATATGCTACTATCTTTGCTTCTTTGCAAGTGGGAAAACTATCAA
monococcum    AACAGAGGATATTATGGCCAATTAACCATGGAACAAATTGGTTAAAGCTTTTCAATATATGCTACTATCTCTGCTTCTTTGCAAGTGGGAAAACTATCAA
              ********************************************************************** *****************************

aestivum      TTTGCATCTTAAACATCACGACCTAGCTATAACCTTTAGTCTTGATTGTGTGCAATCTGCAGCAGAACCCCCATGTACATTTACCTTAATACATCCCACA
urartu        TTTGCATCTTAAACATCACGACCTAGCTATAACCTTTAGTCTTGATTGTGTGCAATCTGCAGCAGAACTCCCATGTACATTTACCTTAATACATCCCACA
monococcum    TTTGCATCTTAAACATCATGACCTAGCTATAACCTTTAGTCTTGATTGTGTGCAATCTGCAGCAGAACTCCCATGTACATTTACCTTAATGCATCCCACA
              ****************** ************************************************* ********************* *********

aestivum      ACAGGCGGTAAACATATTTTTTGACAAAGGAAATATATTAATATTATGGAGATATCAATTACACCCGGCCTCTGCAACAACACAATATCGTAAAAACATT
urartu        ACAGGCGGTAAACATATTTTTCAACAAAGGAAATATATTAATATTATGGAGATATCAATTACACCCGGCCTCTGCAACAACGCAATATCGTAAAAACATT
monococcum    ACAGGCGGTAAACATATTTTTCGACAAAGGAAATATATTAATATTACGGAGATACCAATTACACCCGGCCTCTGCAACAACACAATATCGTAAAAACATT
              *********************  *********************** ******* ************************** ******************

aestivum      ACGGATGCACACAGCCAAAAGATAATAAGAAGAAAAAGAAAAG-----AAAAAAAAGATCCTGCAACAGTGATCAATTCCTCGTAGCTGCAGCACAAACC
urartu        ACGGATGCACACAGCCAAAAGATAATGAGAAGAAAAAGAAAAG----AAAAAAAAAGATCCTGCAACAGTGATCAATTCCTCGTAGCTGCAGCACAAACC
monococcum    ACGGATGCACACAGCCAAAAGATAATAAGAAGAAAAAGAAAAGAAAAGAAAAAAAAGATCCCGCAACAGTGATCAATTCCTCGTAGCTGCAGCACAAACC
              ************************** ****************     ************* **************************************

aestivum      ACCACCTAGACAACACCCGAAGTCCCAATTCTCCAAAAACGACGCCTCCAAGAAGGAAACAGTGCAGAAGCACCGTCGTCGCCCGATCCCAAAGGCGGTA
urartu        ACCACCTAGACAACACCCGAAGTCCTAATTCTCCAAAAACGATGCCTCCAAAAAGGAAACAGTGCAGAAGCACCGTCGTCGCCCGATCCCAAAGACGGTA
monococcum    ACCACCTAGACAACACCCGAAGTCCTAATTCTCCAAAAACGACGCCTCCAAGAAGGAAACAGTGCAGAAGCACCGTCGTCGCCCGATCCCAAAGGCAGTA
              ************************* **************** ******** ****************************************** * ***

aestivum      AACATATGCAATACAAATGCCATTTGCCCCGATAGAATCTTAAGGCTTTCACCTCCTAACATATCTGTTGTTTTTAGTTGTGACTTCATCTGAAAGACCC
urartu        AACATATGCAATACAAATGCCATTTGCCCAGATAGAATCTTAAGGCTTTCACCTCCTAACATATCCGTTGTTTTTAGTTGTGACTTCATCTGAAAGACCC
monococcum    AACATATGCAATACAAATGCCATTTGCCCCGATAGAATCTTAAGGCTTTCACCTCCTAACATATCTGTTGTTTTTAGTTGTGACTTCATCTGAAAGACCC
              ***************************** *********************************** **********************************

aestivum      CTCTTCTAACTAACACTATGGATTGGGTAACATAACTCTGCAGAAAAATAACAAATTAAATCCATTGATTGGTGCATTAACGTTACAAAAATGGCATCAT
urartu        CTCTTCTAACTAACACTATGGATTGGGTAACATAACTCTACAGAAAAATAACAAATTGAATCCATTGATTGGTGCATTAACGTTACAAAAATGGCATCAT
monococcum    CTCTTCTAACTAACACTATGGATTGGGTAACATAACTCTGCAGAAAAATAACAAATTAAATCCATTGATTGGTGCATTAACGTTACAAAAAGGGCATCAT
              ******************************************************* ***************************** ********

aestivum      TAAGGGAAGAATAGAGGAAATTATTGTGGTATCAGCAAAAGAAAAAAGAAAGGGATCCATCGATCAGCAGTAAACCATCTATGTTACTTACCTCAGAAAC
urartu        TAAGGGAAGAATAGAGGAAATTATTGTGGAATCAGCAAAAGAAAAAAGAAAGGGATCCATCGATCAGCAGTAAACCATCTATGTTACTTACCTCAGAAAC
monococcum    TAAGGGAAGAATAGAGGAAATTATTGTGGAATCAGCAAAAGAAAAAAGAAAGGGATCCATCGATCAGCAATAAACCATCTATGTTACTTACCTCAGAAAC
              ***************************** *************************************** ******************************

aestivum      ATAACTATGCATATATACCTTCAGATTGTTGCATGTGTGGGAAGTAATCTTCAAAAAGAAAATGAAAATTAGAAAAAAGTTCAGAGTTAGTGCAGCAAGT
urartu        ATAACTATGCATATATACCTTCAGATTGTTGCATGTGTGGGAAGTAATCTTCAGAAAGAAAATGAAAATTAGAAAAAAGTTCAGAGTTAGTGCAGCAAGT
monococcum    ATAACTATGCATATATACCTTCAGATTGTTGCATGTGTGGGAAGTAATATTCAGAAAGAAAATGAAAATTAGAAAAAAGTTCAGAGTTAGTGCAGCAAGT
              ************************************************ **** **********************************************

aestivum      GACACATCACTTTCGAAATCGCTTGAGTTATTTTTTTACCAGCTTTCCCACACAAAAAAA-GCTGTTACCACAATACCACTAGCAAGCAATGTGAATAAT
urartu        GACACATCACTTTCGAAATCGCTTGAGTTATTTTTTTACCAGCTTTCCCACACAAAAAAAAGTTGTTCCCACAATACCACTAGCAAGCAATGTGAATAAT
monococcum    GACACATCACTTTCGAAATCGCTTGAGTTATC---TTATCAGCTTTCCCACACAAAAAAA-GCTGTTACCACAATACCACTAGCAAGCAATGTGAATAAT
              *******************************    *** ********************* * **** ********************************
```

```
aestivum      TTGTCCACCAGAAGCATGCAAAAAGATACCGACTTCAATACTCCTTTCAGGAAAGAAGCATGAGAATTTTGTATGTCCTTTGTATACATCAAGATTTGTA
urartu        TTGTCCACCAGAAGCATGCAAAAAGATACCGACTTCAATACTCCTTTCAGGAAAGAAGCATGAGAATTTTGTATGTCCTTTGTATACATCAAGATTTGTA
monococcum    TTGTCCACCAGAAGCATGCAAAAAGATACCGACTTCAATACTCCTTTCAGGAAAGAAGCATGAGAATTTTGTATGTCCTTTGTATACATCAAGATTTGTA
              ****************************************************************************************************

aestivum      ACAAAAATAGATTTAGATAGATAGATAGATAG------------------------GGTGCTAAGAGTTACAGATGGCGCCATGGAGGCCTTTTTTTGAA
urartu        ACAAAAATAGATTTAGATAGATAGATAGATAGATAGATAGA-------------AGGGTGCTAAGAGTTACAGATGGCGCCATGGAGGCCTTTTTTTTAA
monococcum    ACAAAAATAGATTTAGATAGATAGATAGATAGATAGATAGATAGATAGATAGATAGGGTGCTAAGAGTTACAGATGGCGCCATGGAGGCCTTTTTTTGAA
              ********************************                        ***************************************** **

aestivum      AACAAATCAAAATTTGAACTTTCTGGTTTGAAAAAATCTGAAAAAAA-TTGTGCAAGTAAACAAGGATGTTATTTGTATGTGTGTAAAATTTCAGGATGA
urartu        AACAAATAAAGATTTGAACTTTCTGGTTTGAAAAAATCTGAAAAAAAATTGTGCAAGTAAACAAGGATGTTATTTGTATGTGTGTAAAATTTCAGGATGA
monococcum    AACAAATCAAAATTTGAACTTTCCGGTTTGAAAAAATCTGAAAAAAAAATGTGTGAGTAAACAAGGATGTTATTTGTATGTGTGTAAAATTTCAGGATGA
              ******* ** ************ ***********************  ****  *********************************************

aestivum      AAAACGTTGAAATGCGATTTTTACAAAAAAGACAAATTTATGGCCTGGGAGGATGAATAGTATCATGTGTTAAAAAGTCTTAGATTTGTCTCTTTTTTGC
urartu        AAAACGTTGAAATGCGATCTTTACAAAAAAGACAAATTTATGGCCTGGGAGGATGAATAGTATCATGTGTTAAAAAGTCTTAGATTTGTCTCTTTTTTGC
monococcum    AAAACGTTGAAATGCGATCTTTACAAAAAAGACAAATTTATGGCCTGGGAGGATGAATAGTATCATGTGTTAAAAAGTCTTAGATTTGTCTCTTTTTTGC
              ***************** **********************************************************************************

aestivum      ACAGCCCTCATTTCAACGTATTTTGTGCTGAAAATTTACACACATGTGTATTATGCCTCATGTATATCTGTGTG--TTTTTTCAGAATTTTTTGAAACAT
urartu        ACAGCCCTCATTTCAACGTATTTTGTCCTGAAAATTTACACACATGTGTATTATGCCTCATGTATATCTGTGT---TTTTTTCAGAATTTTTTGAAACAT
monococcum    ACAGCCCTCATTTCAACGTATTTTGTCCTGAAAATTTACACACATGTGTATTATGCCTCATGTATATCTGTGTTTTTTTTTTCAGAATTTTTTGAAACAT
              ************************** **********************************************   ************************

aestivum      AAAAAATATGAAATTTGACAAAATTCAAAATTTTCAAAACCGAGCTCAATGGAGCTCGGTCTCCAAAGACAATATTCGCCATTGGAGGAGTGGAGGGCCT
urartu        AGAAAATATGAAATTTGACAAAATTCAAAATTTTCAAAACCGAGCTCAATGGAGCTCGGTCTCCAAAGACAATATTCGCCATTGGAGGAGTGGAGGGCCT
monococcum    AGAAAATATGAAATTTGACAAAATTCAAAATTTTCAAAACCGAGCTCAATGGAGCTCGGTCTCCAAAGACAATATTCGCCATTGGAGGAGTGGAGGGCCT
              * **************************************************************************************************

aestivum      ATAGAAGCTAGAAAACAATTAGAATAACACCTT---GTTAACTTATAAGACAAGGAACAATTAATATATTTAAATGCATAAATTACCTTGGGCACTAATC
urartu        ATAGAAGCTAGAAAACAATTAGAATAACACCTT---GTTAACTTATAAGACAAGGAACAATTAATATATTTAAATGCATAAATTACCTTGGACACTAATC
monococcum    ATAGAAGCTAGAAAACAATTAGAATAACACCTTCTTGTTAACTTATAAGACAAGGAACAATTAATATATTTAAATGCATAAATTACCTTGGACACTAATC
              *********************************   ******************************************************* ********

aestivum      AAATCAATCAAAATTAGTAGCTTCAGAACATTTTTAGGACATCAAAAGGGGATGAATAGTACTGGAAAACAACATTTGTATTTT-CCCAAAATATCACCA
urartu        AAATCAATCAAAATTAGTAGCTTCAGAACATTTTTAGGACATCAAAAGGGGATGAATAGTACTGGAAAACAACATTTGTATTTT-CCCAAAATATCACCA
monococcum    AAATCAGTCAAAATTAGTAGCTTCAGAACATTTTCAGGACATCAAAAGGGGATGAATAATACTGGAAAACAACATTTGTATTTTTCCCAAAATATCACCA
              ****** *************************** *********************** ************************* ***************

aestivum      TAAAAATTGCTACAAAATAGGGGGGAGATGGTGACCACGGTAGTTGAGAACAACACCCTGCCTGTACAGAATGGACGTCTGCCACCGCCTATTGACCGCT
urartu        TAAAAATTGCTACAAAATAGGGGGGAGATGGTGTCCATGGTAGTTGAGAACAACACCCTGCCTGTACAGAATGGACGTCTGCCACCGCCTATTGACCGCT
monococcum    TAAAAATTGCTACAAAATAGGGGGGGGATGGTGTCCACGGTAGTTGAGAACAACACCCTGCCTGTACAGAATGGACGTCTGCCACCGCCTATTGACCGCT
              ************************* ******* *** **************************************************************

aestivum      GGCCTCGCCTCTTACAGCAACTTGTGTGTCGACTGTGTCTTGCCATCTCCGCCGCTGGGGAGGGTTTGCTCTTGGAAGACACCGATGTTGGCGATTACCA
urartu        GGCCTCGCCTCTTACAGCAACTTGTGTGTCGACTGTGTCTTGCCATCTCCACCGCTGGGGAGGGTTTGCTCTTGGAAGACACCGATGTTGGCGATTACCA
monococcum    GGCCTCGCCTCTTAGAGCAACTTGTGTGTCGACTGTGTCTTGCCATCTCCGCCGCTGGGGAGGGTTTGCTCTTGGAAGACACCGATGTTGGCGATTACCA
              ************** *********************************** *************************************************
```

```
aestivum      TGGTGGAAACACCAGTCGCGATTTTACATCTGTTGTCCAATGCCATTGAGTTATTATTACCAAACGAATCAGCATGATCACCAATCGCAAAATCATTGAA
urartu        TGGTGGAAACACCAGTCGCGATTTTACATCTGTTGTCCAATGCCATTGAGTTATTATTACCAAACGAATCAGCATGATCACCAATCGCAAAATCATTGAA
monococcum    TGGTGGAAACACCAGTCGCGATTTTACATCTGTTGTCCAATGCCATTGAGTTATTATTACCAAACGAATCAGCATGATCACCAATCGCAAAATCATTGAA
              ****************************************************************************************************

aestivum      TGGAAATATGATGCAAATCTTATCTGCTCATCTCTGAATCCATGTAGGCGAGAAAACTTCAAATATGGCTCTCGGGGTTCCTCCCAACCTTCCACATGGC
urartu        TGGAAATATGATGCAAATCTTATCTGCTCATCTCTGAATCCATGTAGGCGAGAAAACTTCAAATATGGCTCTCGGGGTTCCTCCCAACCTTCCACATGGC
monococcum    TGGAAATATGATGCAAATCTTATCTGCTCATCTCTGAATCCATGTAGGCGAGAAAACTTCAAATATGGCTCTCGGGGTTCCTCCCAACCTTCCACATGGC
              ****************************************************************************************************

aestivum      CGATGGTAGAAGTTAGCACCTTCGACGGCTTCTCTAGGTCGGGCGCAGTGCGACGGTAGGTTCTCGTCAAACACTAACATGCCGCACAGAGGGCAAGAAA
urartu        CGATGGTAGAAGTTAGCACCTCCGACGGCTTCTCTGGGTCGGGCACAGTGCGACGGTAGGTTCTCGTCAAACACTAACATGCCGCACAGAGGGCAAGAAA
monococcum    CGATGGTAGAAGTTAGCACCTCCGACGGCTTCT-TGGGCCGGGCGCAGTGCGACGGTAGGTTCTCGTCAAACACTAACATGCCGCATAGAGGGCAAGAAA
              ********************* *********** * ** ***** ***************************************** *************

aestivum      AGGAGGTGATGGCGAAGCCGGGGTGCGCCGCAAGGAAGAGAAGGACGCAAGGAGGTGAACATGGAGGATCGAGATTCGAGAATATGAAGGAGCAAAAGAG
urartu        AGGAGGTGATGGCGAAGCCGGGGTGCGCCGCAAGGAAGAGAAGGACGCAAGGAGATGAACATGGAGGATCGAGATTCGAGAATATGAAGGAGCAAAAGAG
monococcum    AGGAGGTGATGGCGAAGCCGGGGTGCGCCGCAAGGAAGAGAAGGACGCAAGAAGATGAACATTGAGGATCGAGATTCGAGAATATGAAGGAGCAAAAGAG
              *************************************************** ** ******* *************************************

aestivum      AGGAGTGGAGACAGGGATGTGTTGGTCCCTAATGCCCATCGGCGGAGGCTGGTGGTTGATTTATGTGGTTCGGGAGATTTGGAATTAGAATCGCTTTCCA
urartu        AGGAGTGGAGACAGGGATGTGTTGGTCCCTAATGCCCATCGGCGGAGGCTGGTGGTTGATTTATGTGGTTCGGGAGATTTGGAATTAGAATCGCTTTCCA
monococcum    AGGAGTGGAGACAGGGATGTGTTGATCCCTAATGCCCATCGGCGGAGGCTGGTGGTTGATTTATGTGGTTCGGGAGATTTGGAATTAGAATCGCTTTCCA
              ************************ ***************************************************************************

aestivum      GATTGATTCTTTGGTTCAGTGGAAGAGGAAGCAAACGATCATATCTTTCCCCAGGGTGTGGCTACATATAAACAAAAGTTAATAATACAATTTTTTTT-T
urartu        GATTGATTCTTTGGTTCAGTGGAAGAGGAAGCAAACGATCATATCTTTCCCCAGGGTGTGGCTACATATAAACAAAAGTTAATAATACAAATTTTTTTGT
monococcum    GATTGATTCTTCGGTTCAGTGGAAGAGGAAGCAAACGATCATAGCTTTCCCCAGGGTGTGGCTACATATAAACAGAAGTTAATAATAC--ATTTTTTT-T
              *********** ******************************* ****************************** *************  ******** *

aestivum      GGCTAATGTGGATATGCTGCATGCAAGCTTGCAAGGCTGGGA---ACCGGTCCTCAAACGAAACACATGGGATCGTGGAGATTTGATGACGTGGATGCAT
urartu        GGCTAATGTGGATATGCTGCATGCAAGCTTGCAAGGCTGGGA---ACCGGTCCTCAAACGAAACACATGGGATCGTGGAGATTTGATGACGTGGATGCAT
monococcum    GGCTAATGTGGATATGCTGCATGCAAGCTTGCAAGGCTGGGACAGACCGGTCCTCAAACGAAACACATGGAATCGTGGAGATTTGATGACGTGGATGCAT
              ******************************************   ************************* *****************************

aestivum      GACTGAGGTGAATAGATTGCATGTCAAGATAAATATGATAGTGAAGATGGATCTCTTAGGTACTCCCTCCGGTTCTTTTTAGTTCGCATATAAGATTTGA
urartu        GACTGAGGTGAATAGATTGCATGTCAAGATA----TGATAGTGAAGATAGATCTCTTAGGTACTCCCTCCGGTTCTTTTTAGTTCGCATATAAGATTTGA
monococcum    GACTGAGGTGAATAGATTGCATGTCAAGATAAATATGATAGTGAAGATGGATCTCTTAGGTACTCCCTCCGGTTCTTTTTAGTTCGCATATAAGATTTGA
              *******************************    ************* ***************************************************

aestivum      CTGAAGTCAAGCCTCGTAAAGTTTGACCAACTTTATAGAAAAAAGTATC-AACATTCACAATCTGAAATCAATATCAATAGATGTGTCATGATTTAAAGT
urartu        CTGAAGTCAAGCCTCGTAAAGTTTGACCAACTTTATAGAAAAAAGTATC-AACATTCACAATCTGAAATCAATATTAATAGATGTGTCATGATTTAAAGT
monococcum    CCGAAGTCAAGCCTCGTAAAGTTTGACCAACTTTATAGAAAGAGGTATCTAACATTCACAATCTGAAATCAATGTCAATAGATGTGTCATGATTTAAAGT
              * *************************************** * ***** *********************** * ************************

aestivum      TTCATATTGTATGACTTTAGCA-TGGCAGATGTTGATATTTTTTCATATAAATACGGTCAAACTTTGTGAAATTTGA-TTCAAAGAATACTAATATGCAG
urartu        TTCATATTGTATGACTTTAGCA-TGGCAGATGTTGATATTTTTTTATATAAATACGGTCAAACTTTGTGAAGTTTGACTTCAGAGAATACTAATATGCAG
monococcum    TTCATATTGTATGACTTTAGCAATGACAAATGTTGATATTTTTTCATATAAATACGGTCAAA-TTTGTGAAGTTTGACTTCAGAGAATACTAATATGCAG
              ********************** ** ** *************** ***************** ******** ***** **** *****************
```

```
aestivum        AGTAAAAAGGACCGGCGGAGTATATAGGATGCAAAGATGAAAAAAGCATTTAGGAGCAAATGCAAAAAGTGTGGCGCCATAAAAAAGACGTCCACGGCTA
urartu          AGTAAAAAGGACCGGCGGAGTATATAGGATGCAAAGATGAAAAAAGCATTTAGGAGCAAATGCAAAAAGTGTGGCGCCATAAAAAAGACGTCCACGGCTA
monococcum      AGTAAAAAGGACCAGCGGAGTATATAGGATGCAAGGATGAAAAAATCATTTAGGAGCAAATGCAAAAAGTGTGGCGCCATAAAAAAGACGTCCACGGCTA
                ************* ******************** ********** ******************************************************

aestivum        ATTGATGGATGAACTTCATTGATGTCCGTGGTCTTTAGGCGAGGTGTTCTTATTGGGGTTCCACTGCGTTGTAGAAGTGCGAGGATTTGTATATCTCGGC
urartu          ATTGATGGATGAACTTCATTGATGTCCGTGGTCTTTAGGCGAGGTGTTCTTATTGGGGTTCCACTGCGTTGTAGAAGTGCGAGGATTTGTATATCTCGGC
monococcum      ATTGATGGATGAACTTCATTGATGTCCGTGGTCTTTAGGCGAGGTGTTCTTATTGGGGTTCCACTGCGTTGTAGAAGTGTGAGGATTTGTATATCTCGGC
                ******************************************************************************* ********************

aestivum        TTCCCGTGGGCTTCTCCTCCT 5147
urartu          TTCCCGTGGGCTTCTCCTCCT 5184
monococcum      TTCCCGTGGGCTGCTCCTCCT 5182
                ************ ********
```

Figure S9. Sequence alignment of the 5AL junctions of the 4AL/5AL translocation from *T. monococcum*, *T. urartu* and *T. aestivum*. The 5’ end is centromere-bound and connected to the RLG_Fatima and 3’ end is telomere-bound connected with the *NC332-5A*. MITE DTT_Stolos is highlighted in yellow, and poly(dA:dT) tracts (>6 bp) are highlighted in grey.
